# Wear Prediction of Functionally Graded Composites Using Machine Learning

**DOI:** 10.3390/ma17184523

**Published:** 2024-09-14

**Authors:** Reham Fathi, Minghe Chen, Mohammed Abdallah, Bassiouny Saleh

**Affiliations:** 1 College of Mechanical and Electrical Engineering, Nanjing University of Aeronautics and Astronautics, Nanjing 210016, China; reham.fathy@nuaa.edu.cn; 2 College of Hydrology and Water Resources, Hohai University, Nanjing 210024, China; m.abdallah.hhu@gmail.com; 3 College of Energy and Power Engineering, Nanjing University of Aeronautics and Astronautics, Nanjing 210016, China; 4 Production Engineering Department, Alexandria University, Alexandria 21544, Egypt

**Keywords:** functionally graded composites, magnesium chips, low-cost eggshell reinforcement, wear, machine learning, worn surface

## Abstract

This study focuses on the production of functionally graded composites by utilizing magnesium matrix waste chips and cost-effective eggshell reinforcements through centrifugal casting. The wear behavior of the produced samples was thoroughly examined, considering a range of loads (5 N to 35 N), sliding speeds (0.5 m/s to 3.5 m/s), and sliding distances (500 m to 3500 m). The worn surfaces were carefully analyzed to gain insights into the underlying wear mechanisms. The results indicated successful eggshell particle integration in graded levels within the composite, enhancing hardness and wear resistance. In the outer zone, there was a 25.26% increase in hardness over the inner zone due to the particle gradient, with wear resistance improving by 19.8% compared to the inner zone. To predict the wear behavior, four distinct machine learning algorithms were employed, and their performance was compared using a limited dataset obtained from various test operations. The tree-based machine learning model surpassed the deep neural-based models in predicting the wear rate among the developed models. These models provide a fast and effective way to evaluate functionally graded magnesium composites reinforced with eggshell particles for specific applications, potentially decreasing the need for extensive additional tests. Notably, the LightGBM model exhibited the highest accuracy in predicting the testing set across the three zones. Finally, the study findings highlighted the viability of employing magnesium waste chips and eggshell particles in crafting functionally graded composites. This approach not only minimizes environmental impact through material repurposing but also offers a cost-effective means of utilizing these resources in creating functionally graded composites for automotive components that demand varying hardness and wear resistance properties across their surfaces, from outer to inner regions.

## 1. Introduction

Magnesium (Mg) has enormous potential to reduce emissions in the aerospace and automotive industries due to its beneficial properties such as low density, excellent machinability, effective vibration damping, and recyclability. However, the extensive use of magnesium is hampered by limitations such as poor elastic modulus, insufficient wear resistance, and a high corrosion rate [1]. To overcome these limitations and enhance the properties of Mg, various types of particle reinforcements have been investigated, including borides (such as TiB_2_ and ZrB_2_), carbides (such as B_4_C, SiC, and TiC), oxides (such as Al_2_O_3_ and TiO_2_), and nitrides (such as TiN and BN). By incorporating these reinforcements into the Mg alloy matrix, notable improvements in mechanical properties like tensile strength, hardness, and wear resistance can be achieved. This opens up new avenues for expanding the potential applications of Mg alloys in different industries. However, one key problem connected with these reinforcing particles is their high cost, which limits their practical application in magnesium matrix composites. To address the cost barrier associated with magnesium composites, researchers have pursued two main approaches. The first approach proposes using Mg waste chips generated during the machining process as a potential matrix material. Instead of throwing away these waste chips, they can be collected and processed to create the matrix of Mg composites. By using waste chips as a matrix, raw material costs can be significantly reduced [2]. This approach not only addresses budgetary constraints, but it also promotes sustainability by reusing waste material generated during the machining process, reducing waste and increasing resource efficiency [3,4].

The second approach focuses on using low-cost waste materials as potential reinforcements for Mg alloys. Researchers investigated various waste materials, including agricultural byproducts, industrial waste, and recycled materials, as potential reinforcements [5]. These waste materials are frequently available at low or no cost and can be incorporated into magnesium alloys to improve their mechanical properties. By using these low-cost waste materials as reinforcements, the overall cost of manufacturing Mg composites can be reduced while also diverting waste from landfills. This approach is consistent with sustainable material production processes and promotes waste reduction and resource conservation. Eggshell particles are one of the low-cost waste materials that can be used as particles. This is primarily because using eggshells as reinforcements in Mg alloys provides numerous benefits. They are abundant and inexpensive, allowing for cost-effective manufacturing. The resulting composite is lightweight, which is useful for weight-sensitive applications. Furthermore, eggshells contribute to sustainability by repurposing waste and lowering environmental impact. The presence of calcium carbonate in eggshells improves the mechanical properties of magnesium alloys, making them more suitable for a variety of applications. To show an example, Parande et al. [6] employed eggshell particles with weight fractions of 3, 5, and 7 wt.% to reinforce Mg-Zn composites through powder metallurgy. Their findings found that adding eggshell particles improved microhardness, thermal stability, damping, and yield strength while not significantly changing density. In another related study, Ramanujam et al. [7] used stir casting to create a biocompatible composite of nano eggshell particles reinforced with AZ31 magnesium alloy at varied weight percentages (0, 1, 2, 3, and 4 wt.%). They observed that when the weight fraction of eggshell particles grew, so did their mechanical characteristics. In another related investigation, Srivastava et al. [8] used solid-state multi-pass friction stir processing to create an AZ31B/Si_3_N_4_/eggshell surface composite and characterize its microstructural, mechanical, and tribological performance after one, two, and three tool passes. Their findings revealed that the ultimate tensile stress at one, two, and three tool passes was 357 MPa, 366.5 MPa, and 375.4 MPa, respectively, which was 20–25% above the base alloy. Furthermore, the addition of eggshell particles considerably increased the wear resistance of the produced composites over the base alloy. Recently, Demirdal and Aydın [9] synthesized Mg/eggshell composites with weight fractions of 2.5, 5, and 10 wt.% using powder metallurgy to enhance the wear and corrosion resistance of pure Mg. Their findings indicated that the incorporation of eggshell particles improved wear performance. Nevertheless, eggshell particles had a negative effect on corrosion performance.

Although magnesium matrix composites reinforced with eggshell particles have shown promising results in improving the properties of Mg alloys, there is a common issue in which the weight of the matrix increases as the concentration of the solid phase increases, resulting in the aggregation of particles and potentially weakening the matrix. To address this issue, researchers used functionally graded materials (FGMs) to attain the appropriate matrix characteristics in certain regions, lowering weight while preventing particle aggregation within the matrix [10]. FGMs are composite materials whose component distribution is not uniform but results in a smooth gradient that allows for control and achievement of the desired qualities [11]. This means that parts near to the surface or contact can be built with higher reinforcement concentrations, while interior regions can have a lower concentration to keep the composite lightweight. The progressive change in the composition of FGMs also helps to reduce particle aggregation [12]. Rather than an abrupt concentration change, the gradual gradient allows for a more uniform distribution of reinforcement particles throughout the matrix [13]. This helps to maintain the composite material’s integrity and strength by preventing localized stress concentrations and potential failure sites [14].

When examining the literature, it is evident that there are a lack of studies on functionally graded composites reinforced with bio-waste particles such as eggshell particles. However, a limited number of studies have explored the use of centrifugal casting to produce functionally graded composites with Mg waste chips as the matrix material. One of these studies explored the effect of magnesium waste chips on the microstructure and mechanical properties of graded composites reinforced with B_4_C particles. The results demonstrated increased particle distribution and mechanical properties, particularly in the composites’ outer zone [15]. Another investigation concentrated on using Mg chips to create gradient composites reinforced with SiC particles. Their findings revealed a well-distributed gradient of SiC particles with negligible agglomeration in the AZ91 matrix [16]. Another study examined the use of Mg chips to create gradient composites reinforced with yttrium oxide particles to improve microstructure and mechanical characteristics. The research found that the concentration of yttrium oxide particles had a considerable impact on mechanical qualities, with the outside zone outperforming the middle and inner regions [17]. These studies collectively demonstrate the viability of employing chip waste to create graded composites, providing a viable method for recovering magnesium chips created during machining operations. There are several designs available for statistical analysis to predict the wear rate of homogeneous and graded composites, including Taguchi, the response surface methodology [18], and grey relational analysis [19]; however, each method has limits. The Taguchi technique assumes independent effects of components, neglecting interactions and resulting in erroneous forecasts. The response surface methodology relies on linearity, which may not fully reflect the system, resulting in less accurate predictions. Grey relational analysis assigns equal value to inputs, ignoring variable significance and resulting in biased evaluations. Machine learning (ML) has been used to overcome concerns that have arisen with other statistical approaches. ML, a subset of artificial intelligence (AI), has been used as a foundational and critical component in a variety of industries over the last two decades [20]. The combination of machine learning and material science has two primary benefits: (1) predicting output metrics like wear rate, tensile strength, and hardness; and (2) building complicated non-linear relationships between input factors. Despite the increased demand for data, ML approaches have produced encouraging outcomes with relatively modest datasets. Traditional ML models, such as random forest (RF), decision trees (DTs), artificial neural networks (ANNs), and support vector machines (SVMs), are highly accurate in predicting output measurement parameters. Recently, some studies focused on developing approaches such as the deep artificial neural network (DNN) and deep neural decision trees (DNDTs), which achieved high performance in different applications [21]. Thus, developing and applying a different ML approach is vital in forecasting the wear performance of graded composites, offering viable alternatives to time-consuming and costly experimental methods. On the other hand, tree-based machine learning (TML) models are gaining increasing attention in engineering research due to their swift processing speed, stability, cost-effectiveness in computation, and commendable accuracy. Notable examples include extreme gradient boosting (XGB), light gradient boosting machine (LightGBM), and gradient boosting with categorical feature support (CatBoost) [22,23,24].

Among the TML models, the XGB model exhibits the highest accuracy compared to the other models in predicting wear properties. The LightGBM model predicts wear status using only grinding sound signals, with accuracy exceeding 91% [24]. Also, the LightGBM model exhibits good accuracy relative to other individual and stacking models, such as SVM, RF, linear regression (LR), classification and regression tree (CART), naïve Bayes (NB), and the K-nearest neighbors algorithm (KNN), in terms of estimating tool wear levels [25]. Furthermore, Gao et al. [26] introduced a prediction technique for the material removal rate utilizing acoustic sensing in conjunction with the ensemble XGB model. This approach yielded prediction models characterized by high accuracies. Moreover, some ML algorithms have demonstrated successful predictions of the specific wear rate, achieving coefficient of determination (R^2^) values of 0.996, 0.980, 0.988, and 0.999 for DNN, gradient boosting machine (GBM), RF, and XGB, respectively [27]. The wear behavior of functionally graded composites, combining magnesium matrix waste chips with cost-effective eggshell reinforcements, is a novel area lacking exploration in the current literature, both experimentally and predictively. This study marks a significant advancement in wear behavior prediction, pioneering the integration of magnesium waste chips and eggshell particles into composite development. To forecast wear characteristics, four distinct machine learning algorithms were employed and assessed using a limited dataset from varied test operations. Consequently, the paper aims to fill a research void by examining and comparing the predictive efficacy of XGBoost, LightGBM, DNDT, and DNN models. The study specifically targets predicting wear rates within three zones (inner, middle, and outer) and investigates the effectiveness of these ML models in this context. Notably, the predictive abilities of these models regarding wear rate have not been previously evaluated, rendering this study unique in its assessment of their performance. This research introduces a new application of machine learning models to forecast wear rates in functionally graded magnesium matrix composites reinforced with low-cost eggshell particles, addressing a gap in previous studies, which have not explored such composites with bio-waste reinforcements. The distinctive properties of functionally graded materials, where mechanical characteristics vary across different zones, present a distinctive challenge for predictive modeling. Specifically, the study focuses on predicting wear rates in three distinct zones (inner, middle, and outer) and explores the effectiveness of these ML models in this domain. Notably, the predictive capabilities of these models regarding wear rate have not been previously assessed, making this study novel in its examination of their performance. To attain this goal, the manufacturing process consists of multiple steps. First, the magnesium chips and eggshells are gathered and thoroughly cleaned. They are then pre-mixed before being melted and blended using a stir casting process. The obtained composition is then poured into a centrifugal casting technique, producing the composite with gradient behavior. The wear performance of the samples is tested at sliding speeds ranging from 0.5 to 3.5 m/s, sliding distances ranging from 500 to 3500 m, and applied loads ranging from 5 to 35 N. The worn surfaces in various zones of the graded composites are thoroughly studied in order to gain insight into the prevailing wear mechanism under specified conditions. In addition, four machine learning algorithms are developed to predict the wear performance of the samples, providing a novel approach to assessing the wear performance of graded magnesium composites.

## 2. Materials and Methods

### 2.1. Raw Materials

**Figure 1:**
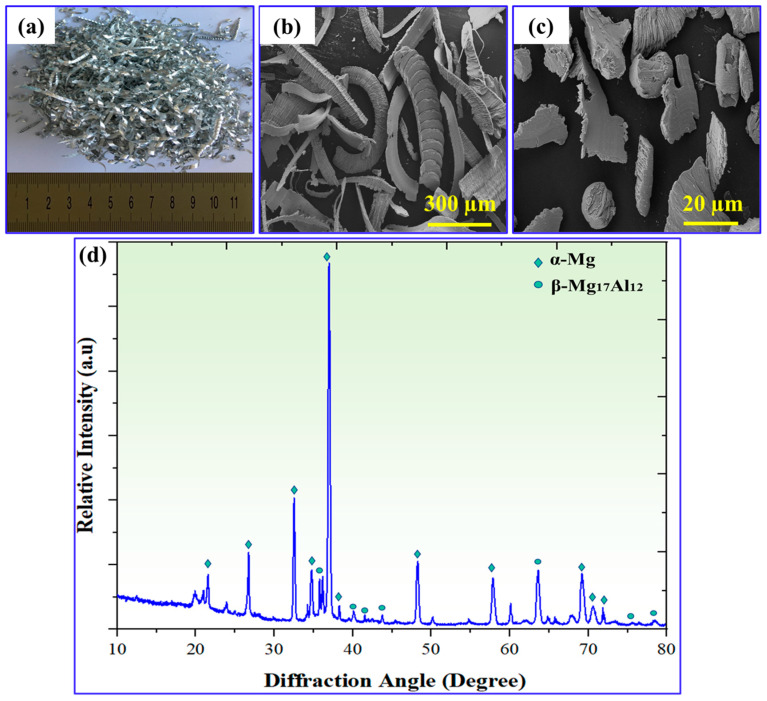
(**a**) Image of AZ91 chips, (**b**) SEM image of the AZ91 chips, (**c**) image of AZ91 chips after crushing, and (**d**) XRD analysis of AZ91 chips after crushing.

Because of its numerous benefits, the AZ91 alloy was selected as the matrix material in this study. This alloy has exceptional mechanical properties and castability, making it the best choice. Its low density, which is one-quarter of steel and two-thirds of aluminum, adds to its attraction [28]. The AZ91 alloy used in this study contained 8.89 wt.% aluminum, 0.72 wt.% zinc, and 0.21 wt.% manganese. It has numerous applications in the aerospace and automotive sectors. The AZ91 magnesium alloy in this study was supplied by Dome Metals Co., Ltd. from Hebi, Henan, China. Table 1 shows the physical and mechanical properties of the AZ91 alloy used in this study. To produce the required chips, dry machining was used on a center lathe machine, eliminating the need for lubricants. This method was chosen to avoid any contamination of the chips that could have occurred if lubricants were used. Following machining, the AZ91 chips were cleaned with ethanol to remove any contaminants left over from the operation. The AZ91 chips were then crushed with a full circle hammer to reduce their size from millimeters to microns. This allowed for efficient mixing with the smaller eggshell particles, which were approximately 15 µm in size. Figure 1 shows an overview of the AZ91 matrix alloy utilized in this study.

**Figure 2:**
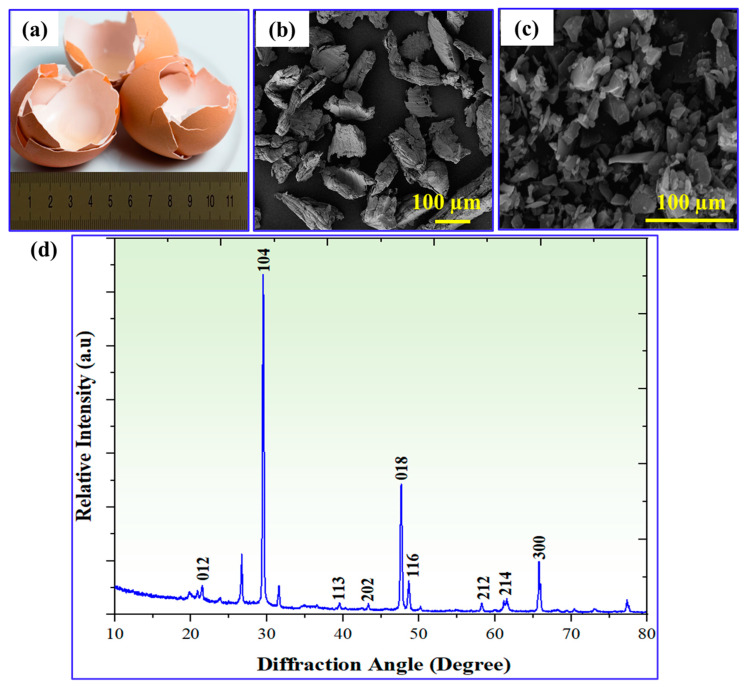
(**a**) Image of eggshells, (**b**) SEM image of the uncarbonized eggshells, (**c**) image of carbonized eggshell particles after crushing, and (**d**) XRD analysis of carbonized eggshell particles after crushing.

In the present investigation, eggshell reinforcement was prepared in a series of steps to ensure that it was suitable for use. The process began with the collection of raw eggshells from local sources in Nanjing, China (Figure 2a). To ensure cleanliness, the eggshells were meticulously cleaned with water and then sun-dried for about 6 h. This step aimed to remove impurities and unpleasant odors associated with eggshells. Once the eggshells dried, they flaked. Using a plastic hammer, the larger eggshells were broken down into smaller flakes. This flaking process made further processing easier and improved the uniformity of the eggshell particles. The eggshell flakes were then further processed into powder form. This was accomplished by using a blender to effectively reduce the flakes to a fine powder. The resulting powder contained smaller particles, which allowed for better dispersion and integration in the graded composite (Figure 2b).

To remove any carbonaceous elements, such as membranes, the powder was carbonized. The powder was exposed to a temperature of 500 °C for 3 h. This carbonization procedure is critical for eliminating any remaining organic debris that may cause porosity in the graded composite. Carbonaceous compounds may cause pores to develop during the sintering process. By heating the powder to high temperatures, these carbonaceous elements evaporate and burn away, effectively lowering the porosity of the eggshell reinforcements. Finally, the carbonized eggshell was crushed with a full-circle hammer. This mechanical process significantly decreased the size of the reinforcement to the micron scale, making it more suitable for use as a reinforcement material. The crushed eggshell particles had an average size of 15 μm, allowing for homogeneous dispersion and integration in the composite matrix (Figure 2c). Previous research has indicated that a weight fraction of 10% is the appropriate number of eggshell particles to generate the necessary gradient within the matrix while preventing the agglomeration of particles [29]. Figure 2d illustrates the XRD patterns of waste eggshell in the form of calcium carbonate (CaCO_3_), with the peaks corresponding to the standard diffraction pattern of CaCO_3_. The presence of a prominent peak, such as the (104) peak, indicates a high concentration of CaCO_3_ in the waste eggshells. This finding suggests that waste eggshells can serve as a natural source of calcium due to their significant CaCO_3_ content.

### 2.2. Composite Fabrication and Characterization Techniques

The AZ91 chip–10 wt.% eggshell graded composite with precise dimensions (outer radius: 90 mm; thickness: 20 mm) was produced in two steps using stir casting and horizontal centrifugal casting. To provide the best outcomes, several strategies were used to address issues such as moisture content, chip oxidation, and particle clustering. To remove moisture, the eggshell particles were first heated at 300 °C for 2 h. This method increased the wettability of the particles when in contact with the molten metal. Afterwards, the crushed AZ91 chips were combined with preheated eggshell particles using a rotary mixer set to 70 rpm for 6 h. This pre-mixing technique efficiently prevented chip oxidation caused by magnesium’s reactivity with oxygen and reduced particle clustering due to the chips’ considerable surface-area-to-volume ratio throughout the alloy reuse procedure. Following the mixing stage, the AZ91 alloy and eggshell particles were moved to a graphite crucible. The chips were gradually melted, beginning at 620 °C and ending at 750 °C. Argon gas was injected into the crucible and ingot chamber to produce a controlled atmosphere, minimizing oxidation and decreasing casting flaws. Once the chips were melted, a mechanical stirrer was used at 250 rpm for 15 min. This stirring operation guaranteed that the eggshell particles were evenly dispersed throughout the AZ91 matrix. Finally, the molten metal, now at 725 °C, was poured into the centrifugal casting mold. The mold revolved at a speed of 1200 rpm, allowing the molten metal to solidify and form the mold. The centrifugal casting technique produced a composite with a significant thickness gradient, particularly in the radial direction.

The microstructure and worn surfaces of the graded samples were investigated using a scanning electron microscope (SEM) equipped with an X-ray energy-dispersive spectrometer. The analysis was performed using the GeminiSEM 300 (Carl Zeiss GmbH, Jena, Germany). Microstructure assessment samples (10 × 10 × 2 mm^3^) were cut using a wire electrical discharge machine. Surfaces were polished in the first step of sample preparation using progressively finer grit emery sheets. The grit sizes employed were 320, 600, 800, 1200, 1500, and 2000, respectively. This method was designed to remove any roughness and defects from the surfaces. To obtain a clean and reflective surface, alumina suspension solutions with various abrasive sizes (2.5 μm, 1 μm, and 0.05 μm) were used as the final polishing step. To obtain the best polishing result, the samples were exposed to each solution separately. An etchant solution containing acetic–picric acid was applied for 5 s to show the boundaries between various microstructural characteristics. This etchant solution targets specific regions, making boundaries more obvious under SEM. Additionally, phase analysis of the raw materials was conducted using an X-ray diffractometer (XRD) with Cu-Kα radiation. The Bruker D8 instrument (Bruker GmbH, Jena, Germany) was employed for this analysis, with a scanning rate of 2.5 degrees per minute.

**Figure 3:**
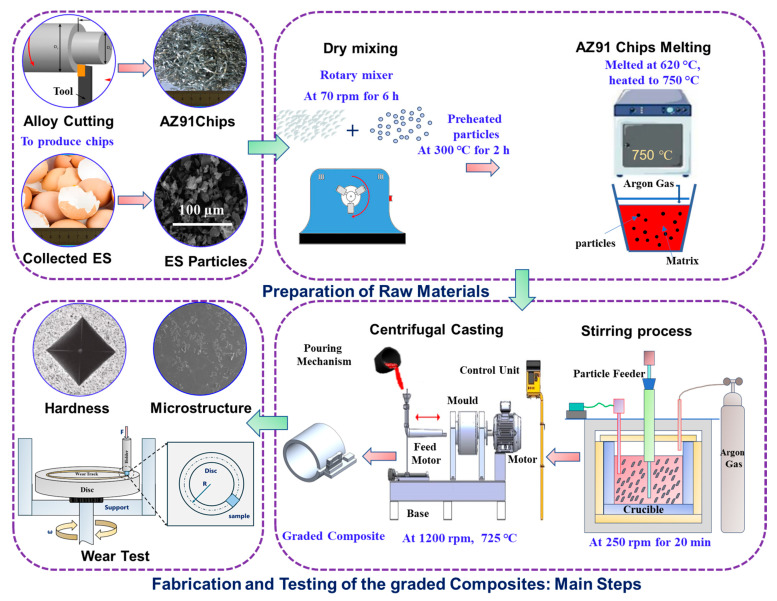
A graphical illustration of the essential steps of the experimental part in this study.

Microhardness was evaluated for 15 s at 100 gf using a digital Vickers hardness tester (HXD-1000TM/LCD, Shanghai Optical Instrument Factory, Shanghai, China). The pin-on-disc wear type machine (TR-20, DUCOM, Ducom Instruments, Bohemia, NY, USA) was used for dry sliding wear testing to assess the wear resistance of graded composites in accordance with the ASTM G99-05 standard [30]. To test pins measuring 10 × 10 × 2 mm^3^, the spinning disc EN31 hardened steel was used. Prior to the wear test, the samples were cleaned and dried with cotton and acetone before being polished with emery sheets of grits 400, 800, 1000, 1500, and 2000, respectively. The wear test was performed at room temperature with varying loads (5 to 35 N) and sliding speeds (0.5 to 3.5 m/s) across sliding distances of 500 to 1000 m. These parameter values were chosen since the wear test offers a comparison value and the ASTM G99 standard [30] does not define specific values for pin-on-disc wear test loads or sliding circumstances. As a result, the loads and sliding conditions used in this study were determined by the target application, pin and disc properties, and previous research. The sliding time in this study accounts for the running-in stage. Additionally, only wear rates are measured during stable circumstances, which are relevant for analyzing the composite’s long-term properties. Following each wear test, the samples were properly cleaned with alcohol and weighed using an accurate digital scale. To determine the wear rate of samples, all wear tests were performed three times to provide a reliable average weight loss result. Figure 3 presents a visual representation outlining the key steps undertaken in the experimental phase of this study.

### 2.3. Data Collection and Processing

In this study, three wear test factors with seven levels were carefully selected, as outlined in Table 2. These factors include the applied load, sliding speed, and sliding distance. By varying these factors across their respective levels, a total of 49 experimental runs were conducted. The experimental values for the three factors (applied load, sliding speed, and sliding distance) at each level, along with the corresponding wear rate responses in different zones (outer, middle, and inner zones), are presented in Table 3. This experimental design enables a systematic investigation of the relationship between various combinations of the applied load, sliding speed, and sliding distance, and the resulting wear rates in different zones. The data provided in Table 3 serve as the foundation for analyzing and discussing the effects of these factors on wear behavior.

**Figure 4:**
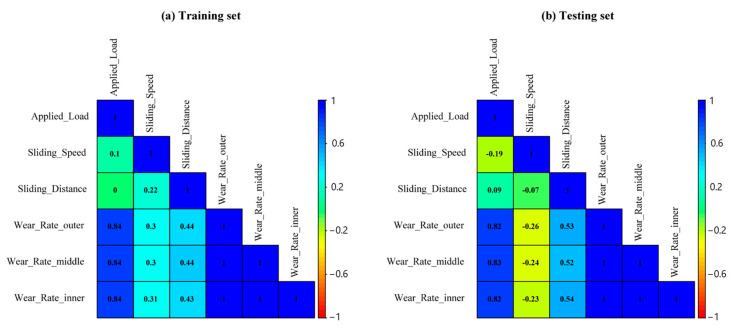
Correlation matrix plots of explanatory variables with wear rate.

To predict wear rate across three zones (outer, middle, and inner), 49 experimental samples were used to train and test four ML models. Three factors including applied load, sliding speed, and distance were considered as explanatory variables to predict wear rate. The first 8 samples in Table 3 were utilized as the test samples while the remaining 41 samples were used in training the models, and their correlation with three wear rate zones is presented in Figure 4. The applied load exhibited the highest correlation (>0.80) with wear rate compared to the other explanatory factors for the training and testing sets. Sliding distance showed a higher correlation than sliding speed for both data sets.

### 2.4. Machine Learning Algorithms

#### 2.4.1. Extreme Gradient Boosting (XGBoost)

The XGBoost model operates on the foundation of a tree learning concept [31]. This algorithm is particularly well-suited for capturing the non-linear features of input variables. Moreover, it belongs to the category of gradient boosting, exhibiting enhanced performance owing to two key improvements: accelerated tree construction speed and a novel tree searching algorithm. This ML model boasts formidable predictive capabilities, leveraging a new regularization technique to mitigate overfitting by introducing an additional term in the loss function [32]. In the context of boosting, the process entails iteratively building models, identifying residuals, and subsequently constructing additional models based on these residuals. This iterative approach aims to maximize the accuracy of the model.

The XGB model is renowned for its versatility, yet its abundance of parameters renders it a complex model. Moreover, to mitigate the risk of overfitting and reduce prediction variability, the careful tuning of hyperparameters is essential. In this study, eight parameters, namely eta, max_depth, n_estimators, learning_rate, gamma, subsample, colsample_bytree, and min_child_weight were used, and their range and optimal values illustrated in Table 4. The range of these parameters was selected according to previous studies [33,34]. The XGB model conducted in R programming language using the xgboost package (https://cran.r-project.org/web/packages/xgboost/index.html, accessed on 8 September 2024).

#### 2.4.2. Light Gradient Boosting Machine (LightGBM)

Chen and Guestrin [35] introduced the LightGBM model as an optimization over the XGBoost model, addressing its limitations. LightGBM leverages several innovative techniques, including the gradient one-sided sampling algorithm (GOSS), the exclusive feature bundling algorithm (EFB), and the leaf-wise growth strategy with depth restrictions. These methods are instrumental in enhancing the model’s classification accuracy.

The LightGBM model demonstrates strong performance and versatility, serving as a wrapper learner for feature selection and effectively addressing classification and regression tasks. Operating as an implementation of the decision tree algorithm, LightGBM can train models to assess the importance of each feature in making predictions. In this study, six parameters were used in the hyperparameter tuning process for LightGBM model, namely max_depth, num_leaves, min_data_in_leaf, feature_fraction, bagging_fraction, and learning_rate. Their range and optimized value are presented in Table 4.

#### 2.4.3. Deep Neural Decision Trees (DNDT)

DNDTs are decision tree models that are implemented in deep learning neural networks (NNs). A particular set of DNDT weightings correlates to a particular decision tree, making the models interpretable [36]. Unlike traditional DTs, DNDT does not require a laborious greedy dividing process since all parameters are optimized concurrently via the adaptive stochastic gradient descent optimization method (Adam). This makes it possible to integrate with bigger NN models for full learning via backward propagation, and it also makes huge-scale computation possible via mini-batch-based training.

Traditional DTs divide features in a demanding and looping manner that might be wasteful at times, but might offer benefits for function choosing [37]. In an effort to boost achievement, recent studies have looked into other methods for developing decision trees, like latent variable-based structured prediction [38]. Using Adam to look for the arrangement of trees and parameters that are DNDT, on the contrary, involves a less complicated method while still being able to produce optimal results when compared to traditional DTs. Furthermore, DNDTs may function with divisions of any cardinality, possibly producing more comprehensible trees than standard DTs, which usually use binary fractions for convenience. In order to facilitate making choices inside DNDTs, the method first uses an indirect separating function to calculate the level of error over each node [39]. The parameters that were used in this study to train the DNDT model are presented in Table 5. This study used the deeptree function from the R package (version 4.2.0) deepdive in order to build the DNDT model (https://cran.r-project.org/web/packages/deepdive/index.html, accessed on 8 September 2024).

#### 2.4.4. Deep Artificial Neural Network (DNN)

DNN is a sophisticated branch of the ANN family that works with complicated problems by combining several hidden layers into its internal architecture [40]. More accurate and physically plausible predictions may be made thanks to this architecture’s notable improvement in capturing input–output interactions [41]. Driven by the networked neurons seen in the human brain, the DNN functions via a two-stage adaptive cycle known as feedforward and backward propagation. However, because the learning rates of different levels differ, having several hidden layers makes the network more complex [42]. There is no standard formula for choosing how many hidden layers and neurons to include in a DNN layer; instead, it is mostly determined by the demands of the task at hand and the properties of the dataset used for training [43]. This study used the deepnet function from the R package deepdive in order to build the DNN model for predicting wear rate, the parameters were optimized using the Adam optimization algorithm, and their values are illustrated in Table 5.

### 2.5. Hyperparameters Techniques

In order to minimize overfitting and attain peak performance, machine learning models are fine-tuned. Finding the optimal parameter values is known as hyperparameter tuning, and it is a crucial step in improving machine learning models. According to Snoek et al. [44], Bayesian optimization (BO) has become a viable technique for hyperparameter tuning, especially in situations when analyzing a collection of parameters requires a lot of time and resources. In this approach, a probabilistic model, usually a Gaussian process, approximates the objective function, which shows the link between ML hyperparameters and how well it performs on a training dataset. The choosing of hyperparameters that show promise for examination in the genuine objective function is guided by this probabilistic approach.

The following is how the algorithm runs:

1. The actual goal function chooses at random and evaluates the initial hyperparameters.
2. Taking into account the starting points, a probabilistic representation of the goal function referred to as the replacement function is built. The Gaussian procedure is frequently used as the surrogate function.
3. By optimizing an acquisition function, a replacement function is used to find the next hyperparameter to assess in the genuine objective function. A common choice for the acquisition function is the Expected Improvement (EI) function.
4. The new assessed point is incorporated in the surrogate function.
5. Steps 2–4 are iterated for a specified number of iterations (N).

The best hyperparameters for machine learning models may be found by efficiently exploring the hyperparameter space using this iterative procedure.

Ten hyperparameter values were selected randomly from the ranges given in Table 4 for the BO algorithm’s first phase. Based on these first observations, a Gaussian process framework was built. By producing posterior distributions of functions with significant uncertainty limits observed further from the points that were sampled as well as small uncertainty bounds seen closer to the sampled locations, the Gaussian process model characterizes the goal function.

Then, at every point across the Gaussian process framework, an ongoing function called Expected Improvement (EI) is calculated. Two factors are evaluated by EI: the first is the predicted change at each point, which is determined through contrasting the Gaussian process model average with the most recent best estimate obtained from the sampled points; the second is the uncertainty of the Gaussian process model at each point, which is determined by uncertainty limits. The point with the highest EI value is chosen to be evaluated in the real objective function, and the output of this procedure is then utilized for updating the Gaussian process model for the next iteration.

The algorithm is prompted to look into fresh domains throughout the early iterations by the EI function, which assigns greater values to locations characterized by high levels of uncertainty. The Gaussian process model’s uncertainty limits become less as the algorithm runs through more iterations and collects more samples, which causes the algorithm to concentrate on areas producing better answers (exploitation). The hyperparameters linked to the best possible outcome are returned by the algorithm following a certain number of iterations, in this case 100. The Bayesian optimization method for this study was carried out using the R package “ParBayesianOptimization” [45].

### 2.6. Model Construction and Evaluation

**Figure 5:**
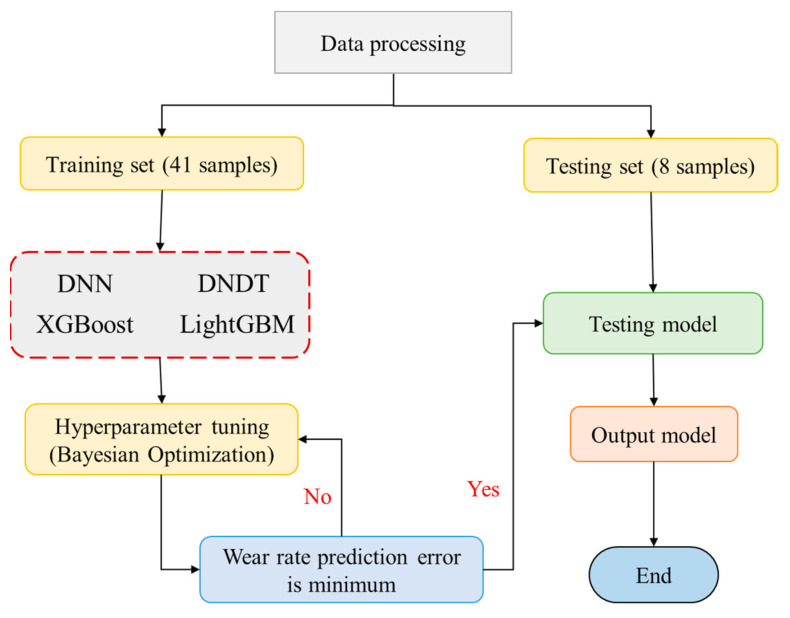
Simplified analytical flowchart depicting the process from data collection to the evaluation of predictive modeling and testing of the predictive model.

In this study, the wear rate across three zones was predicted using the XGBoost, LightGBM, DNDT, and DNN models. Three predictor variables were considered in our work including applied load, sliding speed, and sliding distance for 49 samples. Four ML models were trained using 41 samples, while the remaining samples were used to test the trained models using various statistic metrics. For the XGBoost and LightGBM models, the BO algorithm was used to select the optimal parameter as well as to generalize these models; however, the DNDT and DNN were models optimized using the Adam optimizer. To conduct a comprehensive evaluation of the prediction of wear rate using four ML models including DNN, DNDT, XGBoost, and LightGBM for three types (inner, middle, and outer), three statistical metrics were considered in this study. The statistical metrics that applied in this study, namely R^2^, normalized root mean square error (NRMSE), and the ratio of the RMSE to the measured standard deviation (RSR). The descriptions of these metrics can be found in these studies [22,46,47]. The summary of a simple analytical flowchart illustrating the process from data collection to the evaluation and testing of predictive modeling is shown in Figure 5.

## 3. Experimental Results

### 3.1. Microstructure Analysis

**Figure 6:**
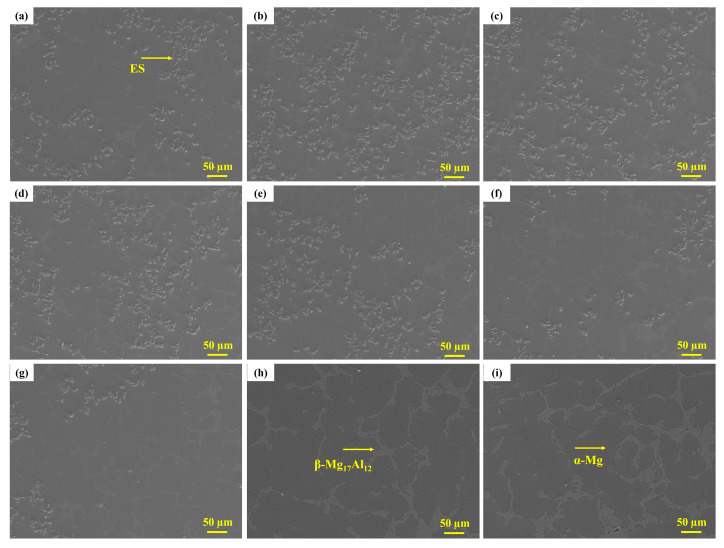
Microstructures of the graded AZ91/ES composite at different distances from the outer to inner surfaces: (**a**) 0.5, (**b**) 1.5, (**c**) 3.5, (**d**) 5.5, (**e**) 6.5, (**f**) 11.5 mm, (**g**) 12.5, (**h**) 15.5, and (**i**) 19.5 mm.

Figure 6 showcases the surface morphology of graded composites within a 20 mm thick composite, where the outer radius measures 90 mm. The micrographs offer valuable insights into the distribution and interaction of the reinforcing eggshell particles within the composite matrix. This evidence supports the successful incorporation of eggshell particles into the composite, contributing to the desired gradient behavior and improved mechanical properties. Notably, the micrographs reveal the presence of primary α-phase from the AZ91 matrix alloy, the β-intermetallic phase of the compound Mg_17_Al_12_ at the grain boundary, and the incorporation of eggshell particles. The presence of the α-phase and β-intermetallic phase further indicates the uniformity and stability of the composite structure. The combination of these phases with the reinforcing eggshell particles enhances the composite’s overall performance, including its mechanical strength, wear resistance, and formability. These observations align with previous studies conducted by various researchers who have reported similar findings [6,48].

When comparing the graded composites reinforced with eggshell particles to previously published results using silicon carbide (3.2 g/cm^3^) or aluminum oxide particles (3.95 kg/mm^3^), it is evident that the eggshell particles exhibit a gradient distribution without any clustering [49,50]. This behavior can be attributed to the density of the eggshell particles (2.17 g/cm^3^) in relation to the density of magnesium (1.81 g/cm^3^). The mobility of particles within the graded composite during the centrifugal casting process is influenced by their densities. Higher density particles tend to migrate towards the outer surface of the composite, while lower density particles (1.81 g/cm^3^) move inward towards the spinning axis. This phenomenon occurs due to the centrifugal force acting on the molten metal and eggshell particles. The higher density eggshell particles (2.17 g/cm^3^) are pushed away from the rotating axis, resulting in the formation of a particle-rich region in the outer zone of the graded composite. Furthermore, to ensure a uniform distribution of particles throughout the matrix, a pre-mixing technique was employed during the preparation of the graded composite. This involved blending the machining chips and eggshell particles before the melting process. This approach effectively homogenized the dispersion of particles within the matrix, preventing agglomeration and promoting a consistent distribution. The combination of the unique density-dependent migration behavior during centrifugal casting and the pre-mixing technique contributes to the successful incorporation of eggshell particles in a graded manner within the composite.

During the casting process, the molten metal is poured into a spinning mold, resulting in the formation of a chill layer with a thickness of 0.5 mm. Due to the temperature difference between the mold (24 °C) and the molten metal (725 °C), the chill layer does not exhibit perfect particle segregation, as seen in Figure 6a. However, this process creates a concentration gradient within the composite, with particles accumulating in the outer zone to achieve a higher particle concentration at a depth of 1.5 mm from the surface, as shown in Figure 6b. Subsequently, the particle concentration gradually decreases across the thickness of the composite in a smooth transition, as shown in Figure 6g. The absence of particles in the inner zone of the centrifugally cast graded composite can be attributed to the specific circumstances and dynamics of the casting process, as shown in Figure 6h,i. Besides, the dendritic structure observed in the inner zone of the composite at positions 15.5 mm 19.5 mm and indicate the absence of particles in this region. This phenomenon is consistent with findings reported in other studies, further supporting the observation that the inner zone of centrifugally cast graded composites tends to lack particles [19,51]. Overall, centrifugal casting involves the rotation of the mold, generating centrifugal forces that drive the molten metal towards the outer regions of the mold. As a result, particles with higher density, such as the eggshell particles (2.17 g/cm^3^), are pushed away from the rotating axis, leading to a particle-rich outer zone. Conversely, the inner zone experiences a relatively lower concentration of particles due to their migration towards the outer regions. These unique characteristics of the centrifugal casting process contribute to the formation of a graded composite, where the particle distribution varies smoothly from the outer zone to the inner zone. This controlled distribution of particles enhances the material’s properties and performance, providing improved mechanical strength and wear resistance.

### 3.2. Hardness Analysis

**Figure 7:**
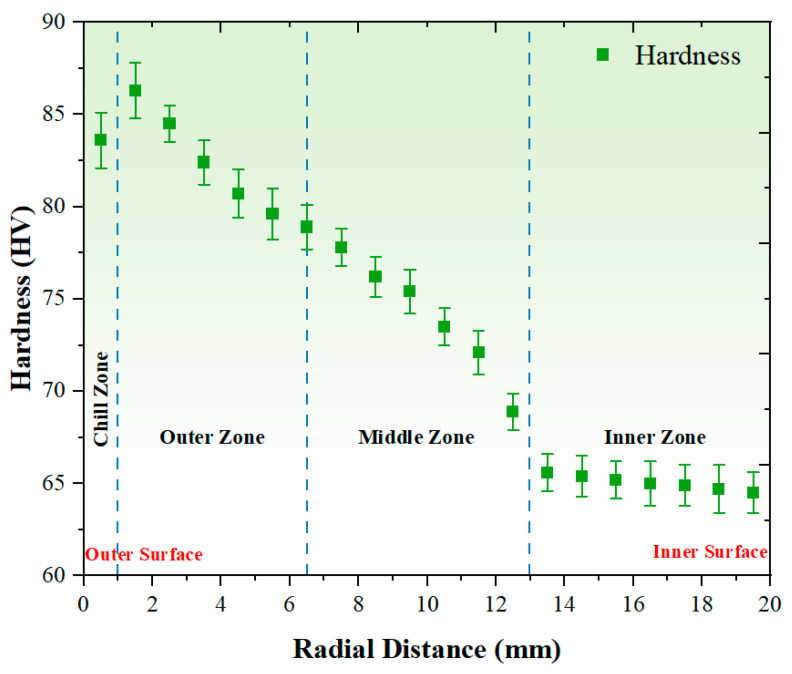
Hardness variation of the graded composite from the outer surface to the inner surface.

Figure 7 illustrates the results of a hardness test conducted on the graded composites, providing further insights into the impact of incorporating eggshell particles into the AZ91 matrix alloy and additional data to support the findings. The insertion of eggshell particles has a significant influence on the overall hardness profile, resulting in a notable increase in hardness due to the finer particle gradient within the matrix alloy. Moreover, in this study, the use of chips effectively dispersed the eggshell particles within the matrix, preventing their agglomeration and further contributing to the observed hardness improvement. The dispersion of particles is crucial for achieving a uniform reinforcement effect and enhancing the mechanical properties of the composite material [52,53]. Examining the hardness trends with increasing thickness, the data shows that initially, as the thickness increases from 0.5 mm to 1.5 mm, there is a slight increase in hardness from 83.6 HV to 86.3 HV, as shown in Figure 7. This initial increase can be attributed to the higher concentration of particles in the outer zone of the composite. However, from 1.5 mm to 6.5 mm, there is a gradual decrease in hardness, with the values decreasing from 86.3 HV to 78.9 HV. Beyond 6.5 mm, the hardness remains relatively stable, with slight fluctuations ranging from 77.8 HV to 64.5 HV as the thickness increases. The main mechanism behind the decrease in hardness with increasing radial distance in the centrifugally cast graded composite can be attributed to the controlled distribution of particles facilitated by the centrifugal casting process. Due to centrifugal forces, particles with higher density, such as the eggshell particles with a density of 2.17 g/cm^3^, are driven away from the rotating axis, leading to a higher concentration of particles in the outer zone of the composite. This higher concentration of particles in the outer zone contributes to the improved hardness at the outer surface of the composite. These trends suggest that the hardness of the composite material initially increases slightly with thickness but tends to stabilize or decrease gradually after reaching a certain threshold, likely influenced by various factors such as the distribution and arrangement of reinforcing particles, as reported by previous studies [54,55].

### 3.3. Wear Rate Analysis

It is well-established that hardness plays a crucial role in improving the wear characteristics of materials. As mentioned previously, the hardness findings of the graded composite revealed that the outer region exhibited higher hardness compared to the middle and inner regions. Furthermore, the utilization of chips during the manufacturing process contributed to the higher hardness values in the outer and middle regions. This can be attributed to the pre-mixing of the chips with the matrix alloy prior to melting, which effectively enhanced the wear resistance in these zones. In the subsequent subsections, the discussion will delve into the effect of wear testing parameters on the wear rate of the graded composite in different zones.

#### 3.3.1. Effect of Applied Load

**Figure 8:**
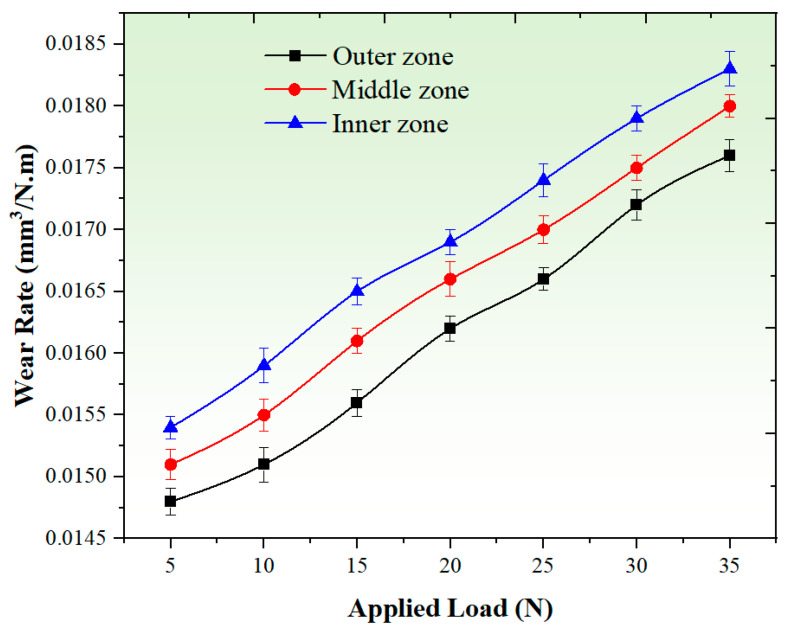
Effect of applied load on the wear rate of the graded composite in various zones under sliding conditions of 3.5 m/s and 1500 m.

Figure 8 provides insights into the effect of different applied loads (ranging from 5 N to 35 N) on the wear rate of the graded composite across various zones. The observed increase in wear rate with higher applied loads can be attributed to the elevated friction between the pin surface and the disc. As the load increases, the contact pressure at the interface intensifies, resulting in more severe mechanical interactions and material removal during wear. The higher contact pressure leads to increased abrasive and adhesive wear, contributing to a higher wear rate. Additionally, the rise in interfacial temperature induced by the increased applied load can have adverse effects on the material properties of the composites. The elevated temperature can cause softening of the material, reducing its strength and wear resistance. This softening effect may further contribute to the increased wear rate observed at higher applied loads [56]. The declining strength of the composites under higher loads contributes to the accelerated wear rates observed. However, it is important to note that the wear rate in the outer zone of the graded composites exhibits a relatively lower increase with the rise in applied load, as depicted in Figure 8. This can be attributed to the increased hardness in the outer region due to the presence of hard particles. These hard particles within the matrix act as barriers, reducing the influence of the applied load during the wear testing. By mitigating plastic deformation, they significantly decrease the wear rate. The wear rate levels of the graded composite at an applied load of 5 N were observed to be 0.0148 mm^3^/N.m, 0.0151 mm^3^/N.m, and 0.0154 mm^3^/N.m for the outer, middle, and inner zones, respectively. On the other hand, at an applied load of 35 N, the wear rate levels for the graded composite were found to be 0.0176 mm^3^/N.m, 0.018 mm^3^/N.m, and 0.0183 mm^3^/N.m for the outer, middle, and inner zones, respectively. Nonetheless, for the graded composites as a whole, the wear rate increases rapidly as the applied load varies from 5 N to 35 N. At the lowest applied load of 5 N, the wear rate in all zones of the graded composites remained low. This is due to the smaller contact stresses exerted on the sliding pin, resulting in reduced material removal during wear. Conversely, the highest wear rate in all zones is observed at the highest applied load of 35 N. This is primarily attributed to the high contact pressure, leading to an increase in temperature. The elevated temperature contributes to the cohesion of the pin material on the rotating disc, further accelerating material loss. According to Archard’s model, the wear resistance of the produced composite continuously decreases with an increase in the contact pressure between the sample and the disc [57,58].

**Figure 9:**
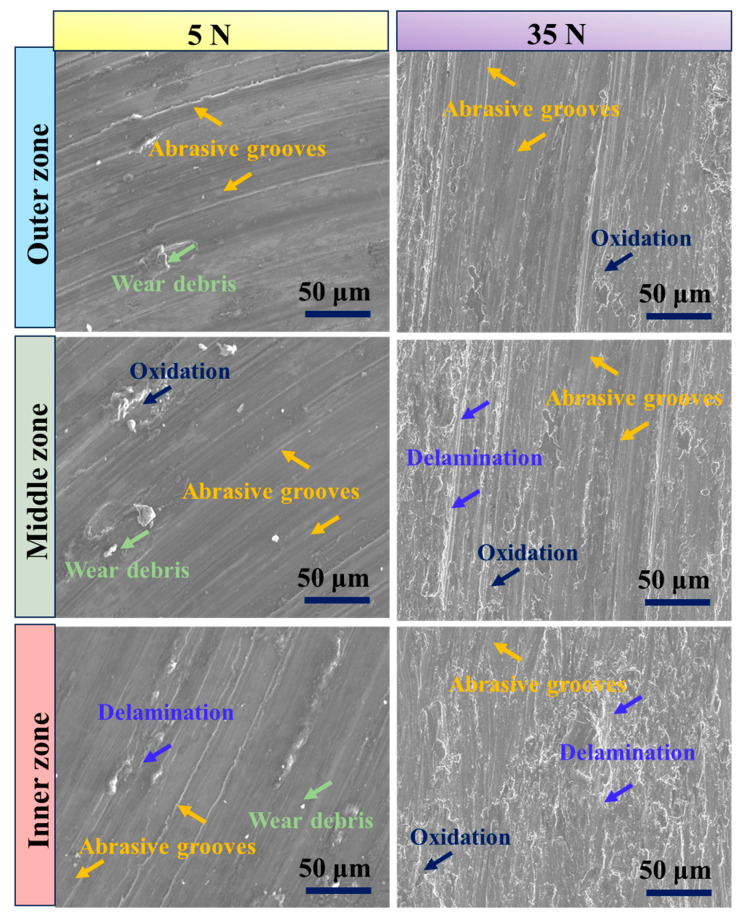
Worn surfaces of the graded composite in different regions under various applied loads (5 N and 35 N) with constant sliding conditions of 3.5 m/s and 1500 m.

Figure 9 presents the SEM images of the worn surfaces at 5 N and 35 N for the different regions (outer, middle, and inner) of the graded composite under sliding conditions of 3.5 m/s and 1500 m. The SEM images provide insights into the wear characteristics of each region. In the SEM images, it is evident that the outer region exhibits better wear resistance compared to the other regions. This is illustrated in Figure 9, where the worn surface of the outer region shows fewer grooves and less degradation. The improved wear resistance in the outer region can be attributed to the highest concentration of eggshell particles present in this region. These eggshell particles act as reinforcing agents, enhancing the hardness and wear resistance of the composite material. As the surface distance moves towards the inner region of the composite, the concentration of eggshell particles within the matrix decreases. This reduction in the concentration of reinforcing particles leads to an increase in the wear rate, causing a change in the wear mechanism from mild to severe wear. This transition is depicted in Figure 9, where the worn surfaces of the middle and inner regions exhibit a higher number of grooves and ploughing. Notably, the absence of eggshell particles in the inner region contributes to its higher wear susceptibility. Without the reinforcing particles, the inner region experiences increased material removal, resulting in the formation of more grooves and ploughing on the worn surface. Thus, the presence and concentration of eggshell particles play a crucial role in determining the wear resistance of each region, with the outer region benefiting from their highest concentration [59,60]. The observed wear mechanisms range from mild in the outer region to severe in the inner region, highlighting the importance of particle distribution and reinforcement in controlling the wear performance of the graded composite.

#### 3.3.2. Effect of Sliding Speed

**Figure 10:**
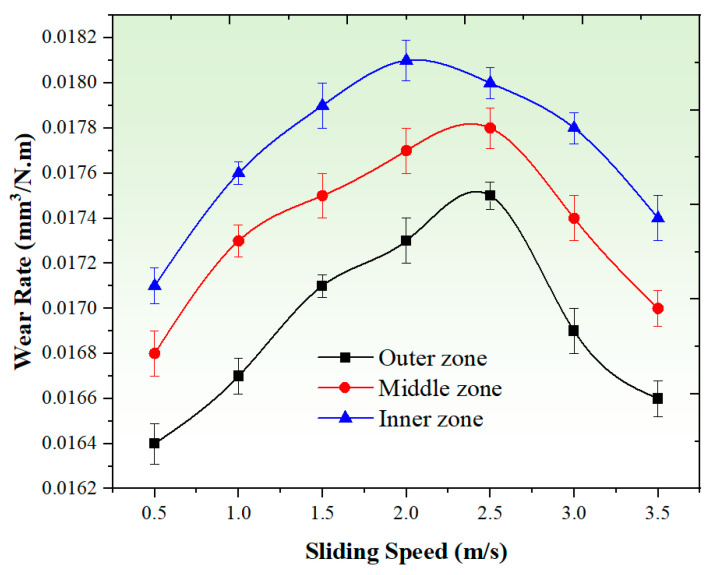
Effect of sliding speed on the wear rate of the graded composite in different regions under sliding conditions of 25 N and 1500 m.

Figure 10 presents the influence of different sliding speeds (ranging from 0.5 m/s to 3.5 m/s) on the wear rate of the graded composite in various zones. The graph reveals important trends regarding the relationship between sliding speed and wear rate. Initially, as the sliding speed increases from lower speeds to 2.5 m/s, the wear rate of the samples shows a corresponding increase. This can be attributed to the contact time between the sample and the rotating disc. When sliding at lower speeds, the extended contact time results in increased contact pressure at the touchpoint, leading to a higher wear rate for the graded composite. Additionally, the shear strain also rises with sliding speed until reaching 2.5 m/s, contributing further to a decrease in wear resistance. However, beyond the sliding speed of 2.5 m/s, a notable decrease in the wear rate is observed, indicating a transition in the wear mechanisms. This shift can be explained by the contact behavior between the composite material and the counter-surface. At lower sliding speeds, the prolonged contact time allows for more material removal and wear. In contrast, higher sliding speeds generate greater relative velocities between the surfaces, resulting in more severe frictional forces and increased energy dissipation. As a consequence, more material is subjected to abrasion and removal, leading to higher wear rates [1,61].

Interestingly, it has been observed that at sliding speeds above 2.5 m/s, the wear rate of the graded composite samples starts to decrease. The phenomenon of the wear rate increasing with the rising sliding speed and then decreasing after reaching 2.5 m/s can be explained by several additional factors. Firstly, as the sliding speeds increase, abrasive wear becomes more pronounced, with harder counter-surface asperities intensifying wear, potentially causing a decrease in the wear rate beyond 2.5 m/s. Secondly, the elevated temperatures resulting from increased friction at higher sliding speeds can alter material properties like hardness and wear resistance. Initially, the wear rate may rise due to softened surfaces, but beyond 2.5 m/s, thermal effects could stabilize or change material properties in a way that reduces wear. Thirdly, debris at lower sliding speeds might contribute to higher wear rates. However, as speed increases, the formation of a debris layer between surfaces can reduce direct contact and friction, potentially leading to decreased wear rates, especially beyond 2.5 m/s. Lastly, higher sliding speeds can induce more significant plastic deformation in the material initially, which adds to an increase in wear. Yet, beyond a certain speed threshold, the material’s deformation mechanisms may shift, potentially resulting in a decrease in the wear rate. These observations regarding the decrease in wear rate at higher sliding speeds and the formation of a protective layer align with findings from previous studies, thus supporting the notion of a transition in wear mechanisms and the influence of a protective layer on the wear behavior of the graded composite [60,62].

**Figure 11:**
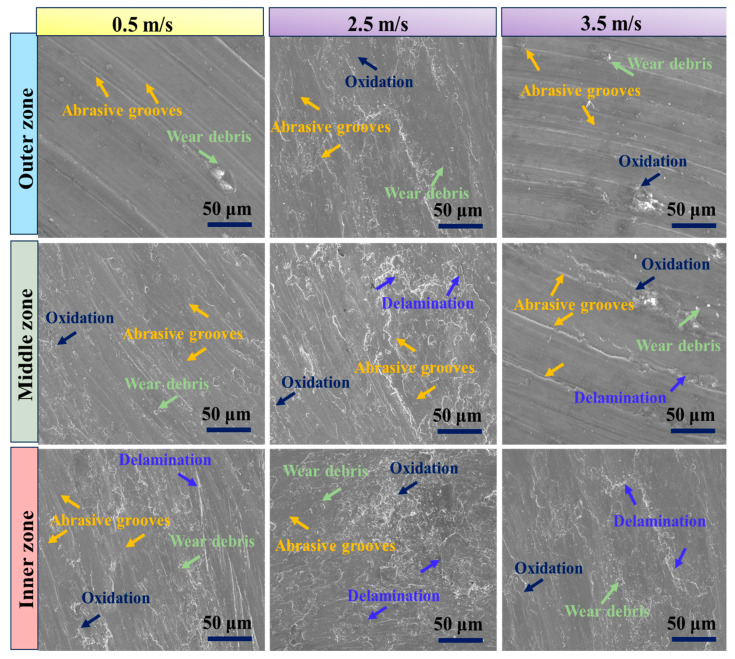
Worn surfaces of the graded composite in different regions under various sliding distance (0.5 m/s, 2.5 m/s, and 3.5 m/s) with constant sliding conditions of 25 N and 1500 m.

The worn surfaces of the graded composite were analyzed under a constant applied load of 25 N and a sliding distance of 1500 m, at three distinct sliding speeds: 0.5 m/s, 2.5 m/s, and 3.5 m/s. Figure 11 provides valuable insights into the wear behavior observed at these sliding speeds. At the lower sliding speed of 0.5 m/s, the SEM images in Figure 11 demonstrate a relatively modest wear rate. The worn surface displays a limited number of grooves and ploughing, indicating that ultra-mild wear is the dominant mechanism. This wear behavior is characterized by minimal material removal and surface damage. As the sliding speed increases to 2.5 m/s, a significant change in wear characteristics is observed. The SEM images reveal a notable increase in the number of deep grooves, ploughing, and delamination occurring parallel to the sliding direction. This shift signifies a transition in the wear mechanism from ultra-mild to severe wear. Furthermore, the presence of wear-induced craters further corroborates this change in wear behavior. However, at the higher sliding speed of 3.5 m/s, the number of grooves on the worn surface decreases significantly, as depicted in Figure 11. This transition in the wear mechanism from severe to mild wear aligns with the observed decrease in wear rate. At higher sliding speeds, the predominant wear mechanism may shift from abrasive wear to a combination of abrasive and adhesive wear. The increased sliding speed facilitates the formation of a smoother and more polished surface, thereby reducing abrasive interactions and wear [61]. The change in wear behavior, as indicated by the SEM images, aligns with the trend observed in Figure 10, where the wear rate initially increases and then decreases as the sliding speed increases. The decrease in wear rate at a sliding speed of 3.5 m/s can be attributed to several factors. These include a shift in wear mechanisms, a reduction in the number of grooves on the worn surface, and the transition from predominantly abrasive wear to a combination of abrasive and adhesive wear at higher sliding speeds. These findings are consistent with previous research conducted on composites with a magnesium matrix and are in agreement with the conclusions drawn from earlier studies [8,63].

#### 3.3.3. Effect of Sliding Distance

**Figure 12:**
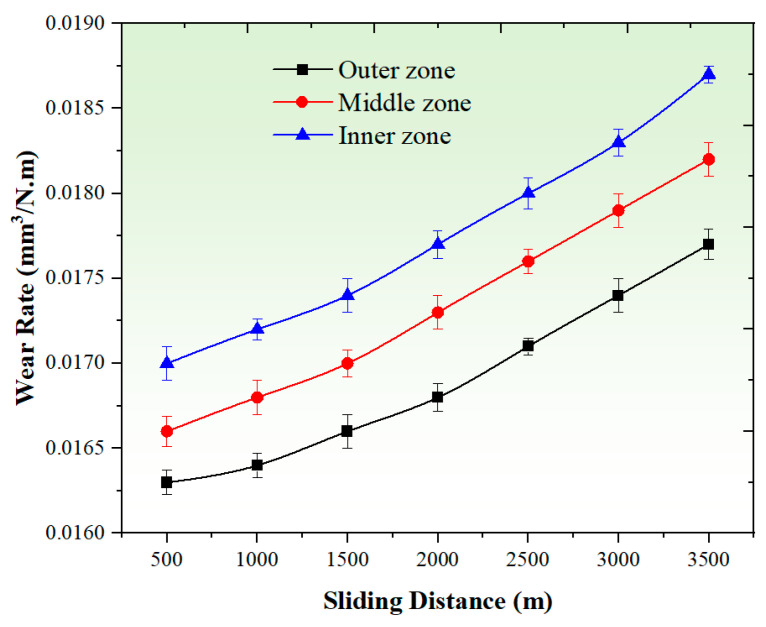
Effect of sliding distance on the wear rate of the graded composite in various zones under sliding conditions of 25 N and 3.5 m/s.

Figure 12 illustrates the impact of varying sliding distances, ranging from 500 m to 3500 m, on the wear rate of the graded composite in different zones. The observations indicate a significant decrease in the wear resistance of the composite as the sliding distance extends from 500 m to 3500 m. This increase in wear rate can be attributed to the heightened friction between the sample and the rotating disc, resulting from the prolonged sliding distance. A longer sliding distance leads to an extended duration of contact and increased rubbing, intensifying the wear process and accelerating material removal from the graded composite. Furthermore, the rise in friction surface temperature at high sliding distances, such as 3500 m, can significantly impact the properties of the graded composite and contribute to a substantial increase in wear rate. However, the presence of eggshell particles within the composite matrix plays a crucial role in mitigating the effect of sliding distance on the worn surface. As depicted in Figure 12, the wear rate values of the graded composite exhibit a slight increase as the sliding distance increases from 500 m to 1500 m. However, beyond a sliding distance of 1500 m, there is a notable rise in the wear rate, indicating a transition in wear mechanisms at this specific sliding distance. The transition observed at 1500 m suggests that the wear behavior of the graded composite undergoes a change beyond this distance. Further exploration of the wear mechanisms involved in this transition can be conducted through the analysis of Figure 12.

**Figure 13:**
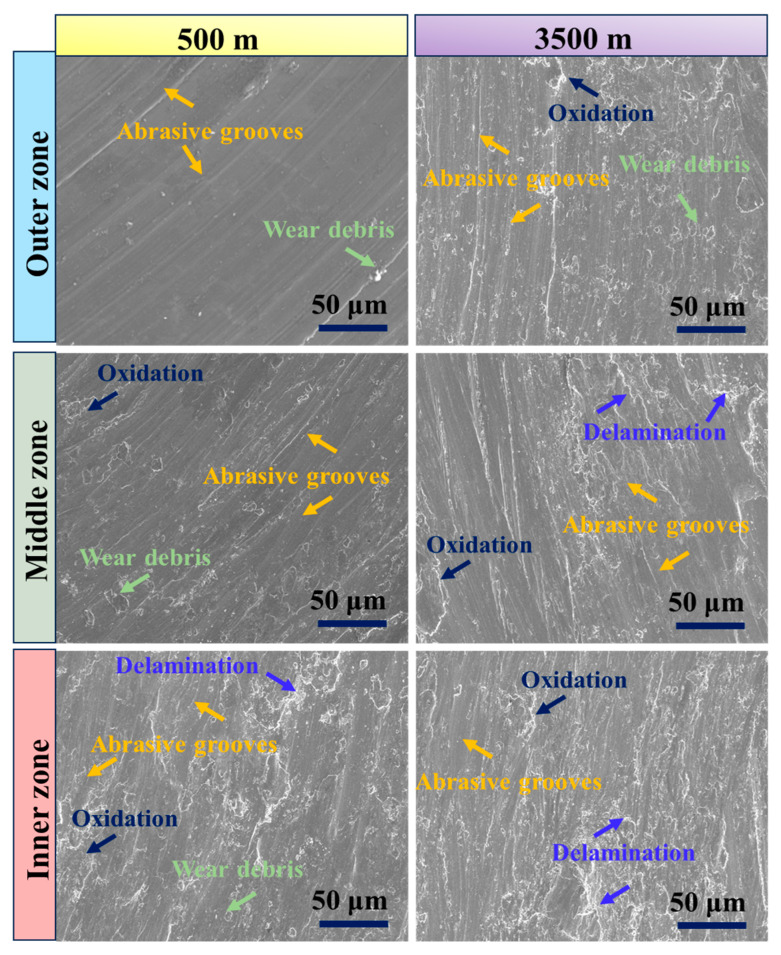
Worn surfaces of the graded composite in different regions under various sliding distances (500 m and 3500 m) with constant sliding conditions of 25 N and 1500 m.

Figure 13 presents SEM images of the worn surfaces of the graded composite in different zones (500 m and 3500 m) under constant wear testing conditions of 25 N and 3.5 m/s. These images provide insights into the wear behavior of the composite at different sliding distances. At the lower sliding distance of 500 m, the SEM images reveal a worn surface with a lesser number of grooves and ploughing, as depicted in Figure 13. This observation suggests a low material removal rate and indicates that ultra-mild wear is the main wear mechanism operating at this sliding distance for the graded composite. Ultra-mild wear is characterized by minimal surface damage and material removal, resulting in a relatively smoother worn surface. In contrast, at the higher sliding distance of 3500 m, the SEM images exhibit distinct changes in the worn surface of the samples. Delamination, larger grooves, and ploughing can be observed, as indicated in Figure 13. These features signify a shift in the wear mechanism of the graded composite at higher sliding distances. The increase in delamination, deeper grooves, and more pronounced ploughing highlights a more severe wear behavior, with greater material removal and surface damage. The changes in wear mechanism observed at different sliding distances suggest that the wear behavior of the graded composite evolves as the sliding distance increases. The transition from ultra-mild wear at 500 m to more severe wear at 3500 m is evident from the SEM images, indicating a progressive change in the wear mechanisms with sliding distance. These results are in line with previous research conducted on composites with a magnesium matrix and are consistent with the conclusions drawn from earlier studies [51,64].

**Figure 14:**
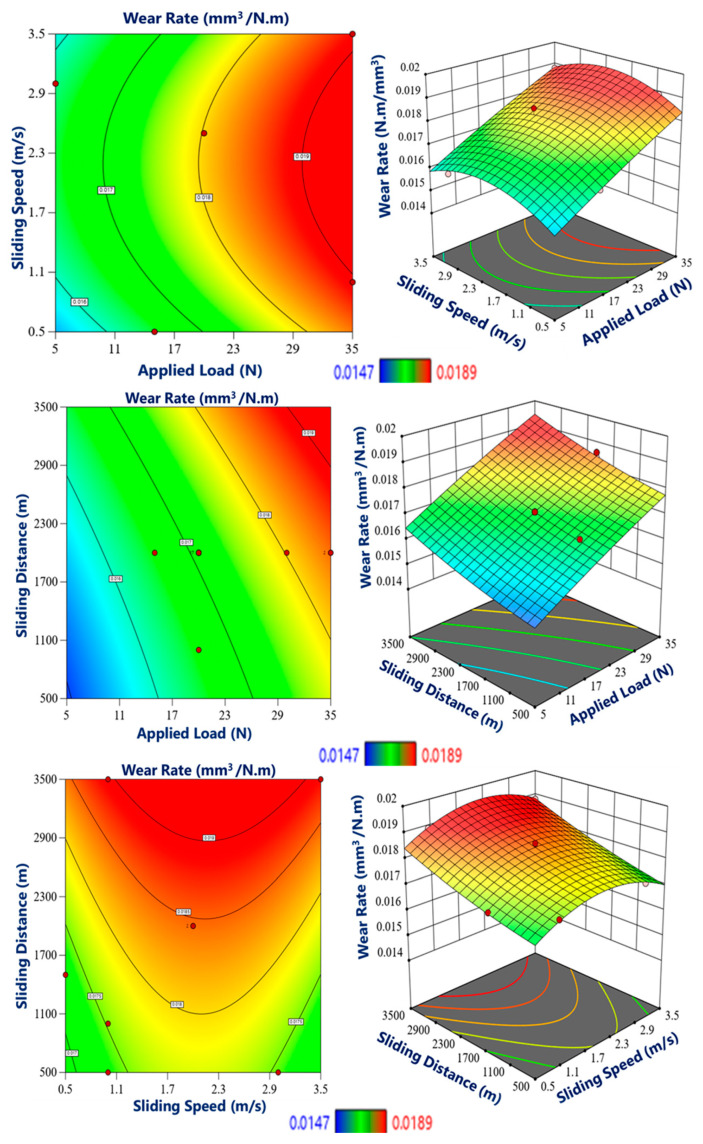
Effect of the interaction between wear parameters on the wear rate of the graded composite in the outer zone.

**Figure 15:**
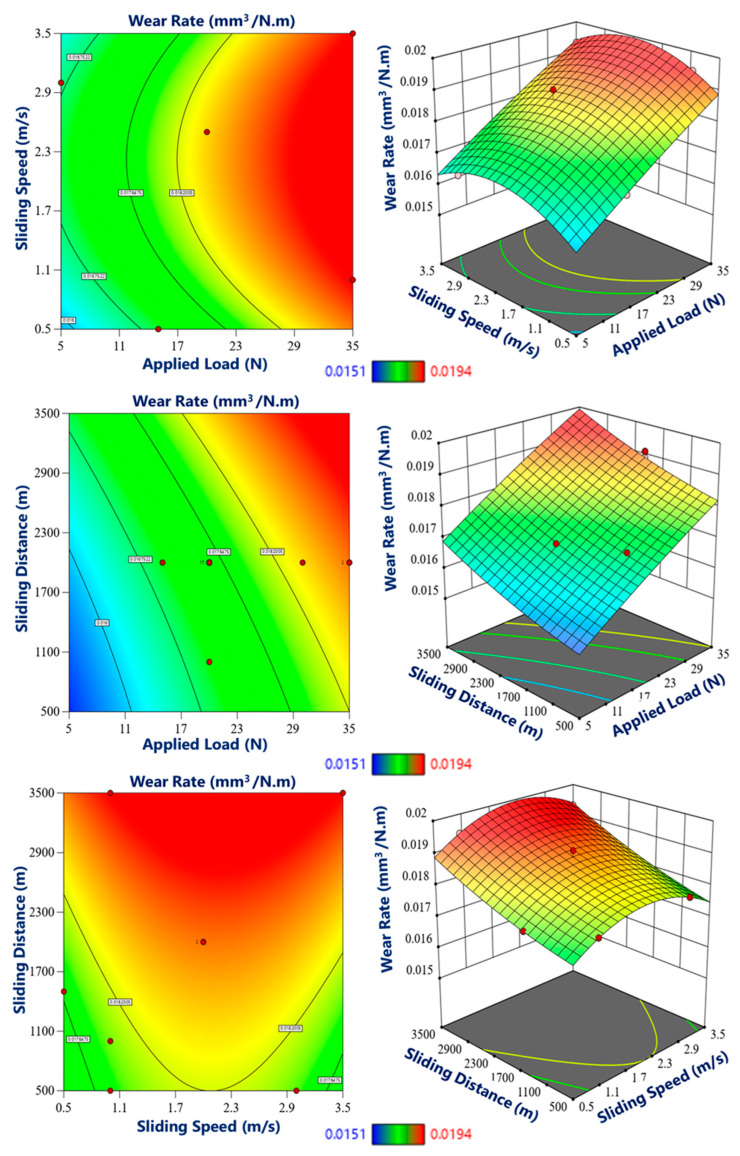
Effect of the interaction between wear parameters on the wear rate of the graded composite in the middle zone.

**Figure 16:**
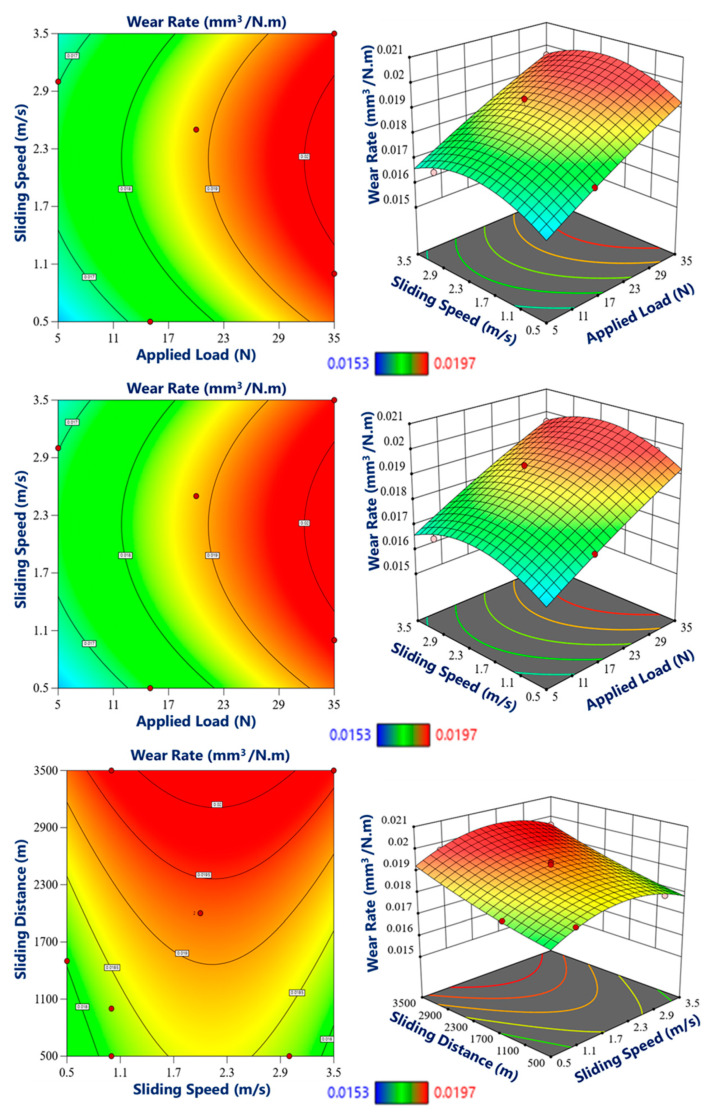
Effect of the interaction between wear parameters on the wear rate of the graded composite in the inner zone.

Figure 14, Figure 15 and Figure 16 illustrate the plots of the main effects, interaction plots as well as the 3D plots of the influence of the various testing parameters on the wear rate of the graded composite in different zones. The main effect graphs and interaction graphs have no noticeable effect if the line for a specific parameter is near horizontal. In comparison, the most crucial influence of a parameter for which the line is the highest incline. Therefore, based on the below figures, it is clear that the most important effects on the wear rate of the graded composites are caused by the sliding speed and applied load while the sliding distance shows less effect on wear rate. In the outer region, characterized by higher hardness and improved wear resistance, the wear rate was found to be significantly lower compared to the other regions, as shown in Figure 14. This can be attributed to the enhanced hardness, which provides a protective barrier against wear and reduces the material loss during sliding contact. The wear rate in the outer region exhibited a substantial reduction, indicating superior wear resistance.

Moving towards the middle and inner regions, which possessed relatively lower hardness values, the wear rate was comparatively higher, as shown in Figure 15 and Figure 16. The decreased hardness in these regions resulted in reduced resistance to wear, leading to increased material loss during the wear testing. However, it is important to note that despite the relatively higher wear rate, the graded composite still exhibited improved wear performance compared to the base material without the incorporation of eggshell particles. Furthermore, the wear testing parameters, such as the applied load, sliding distance, and sliding speed, played a significant role in influencing the wear rate in different zones of the graded composite. Higher applied loads and longer sliding distances resulted in increased wear rates across all regions. Similarly, higher sliding speeds also contributed to accelerated wear. These observations are consistent with other studies [51,65].

#### 3.3.4. Wear Mechanisms

In tribology systems, wear mechanism maps have become valuable tools for understanding and optimizing industrial applications. These maps provide a visual representation of the relationship between sliding speed and applied load, allowing for the identification of optimal conditions for a specific tribo-system. Typically, wear mechanism maps are two-dimensional graphs that display the relative sliding speed and applied load, although other coordinate combinations can be used depending on the application [56,66]. Figure 16 illustrates the wear mechanism maps for the graded composite in different zones, based on the wear rate results and SEM observations of the worn surfaces presented in this study. These maps enable a comprehensive understanding of the dominant wear mechanisms and their transitions across different operating conditions. Figure 16 clearly illustrates a discernible trend in which the zones dominated by ultra-mild and mild wear mechanisms decrease in size as the radial distance from the internal surface to the external surface increases. Conversely, the zones characterized by moderate and severe wear mechanisms expand. This trend can be attributed to the higher concentration of eggshell particles present in the graded pins, which increases with radial distance.

**Figure 17:**
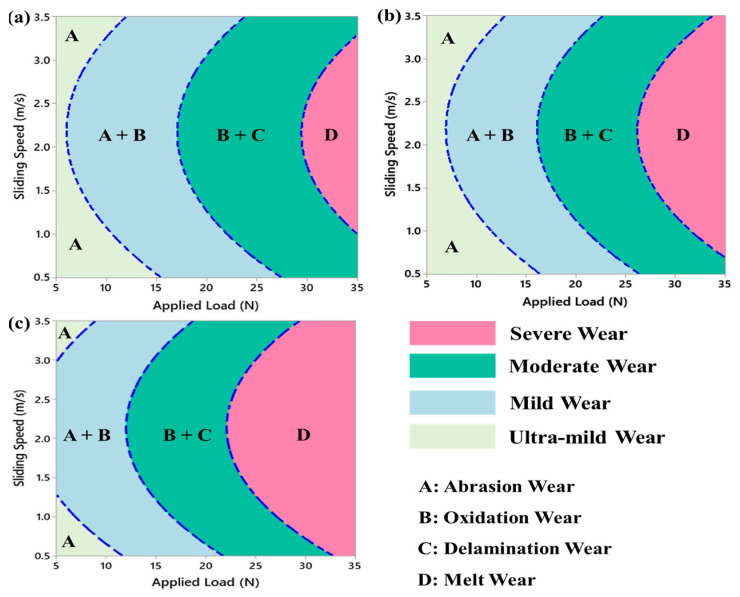
Wear mechanism maps illustrating the wear behavior of the graded composite in different regions: (**a**) outer region, (**b**) middle region, and (**c**) inner region.

Based on SEM examinations, the outer zone of the graded composite exhibits abrasion (ultra-mild wear) as the dominant wear mechanism at low applied loads up to 15 N and moderate sliding speeds ranging from 2 m/s to 2.5 m/s. As the applied load and sliding speed increase within a specific range, the wear mechanism transitions from ultra-mild wear (abrasion) to mild wear (a combination of abrasion and oxidation), depicted in Figure 17a. At specific sliding speeds and moderate applied loads, the dominant wear mechanisms in the graded composite transition from mild wear to moderate wear, characterized by oxidation and delamination. This transition is attributed to the increase in interface temperature between the sample and the rotating disc, which is caused by higher applied loads and intermediate sliding speeds. The elevated temperature induces thermal softening and plastic deformation of the AZ91 matrix alloy. Consequently, the wear rate of the graded composite increases, leading to the prevalence of the severe wear mechanism, as depicted in Figure 17a. When the concentration of eggshell particles is higher, as observed in the outer and middle zones, the transition to a different wear mechanism requires a simultaneous increase in both sliding speed and applied load. This is evident in Figure 17b, where the transition from ultra-mild wear to mild wear or from mild wear to moderate wear occurs within specific ranges of sliding speed and applied load. The increase in both parameters is necessary due to the enhanced wear resistance imparted by the higher concentration of particles. In contrast, in the inner zone (Figure 17c), where the concentration of eggshell particles is absent, the transition to a different wear mechanism primarily occurs by increasing either the applied load or the sliding speed. This indicates that in the absence of eggshell particle concentration, a change in wear mechanism can be achieved by adjusting only one of the parameters. The increase in wear resistance resulting from the higher concentration of eggshell particles is reflected in these observations. The need for simultaneous increases in sliding speed and applied load to trigger a wear mechanism transition in the outer zones suggests that the higher particle concentration contributes to improved wear resistance [16,56]. Table 6 provides a summary of the observed wear mechanisms in the graded composite in different zones under various combinations of applied load and sliding speed.

## 4. Discussion of Machine Learning Models

### 4.1. ML Models’ Performance in Inner Zone

**Figure 18:**
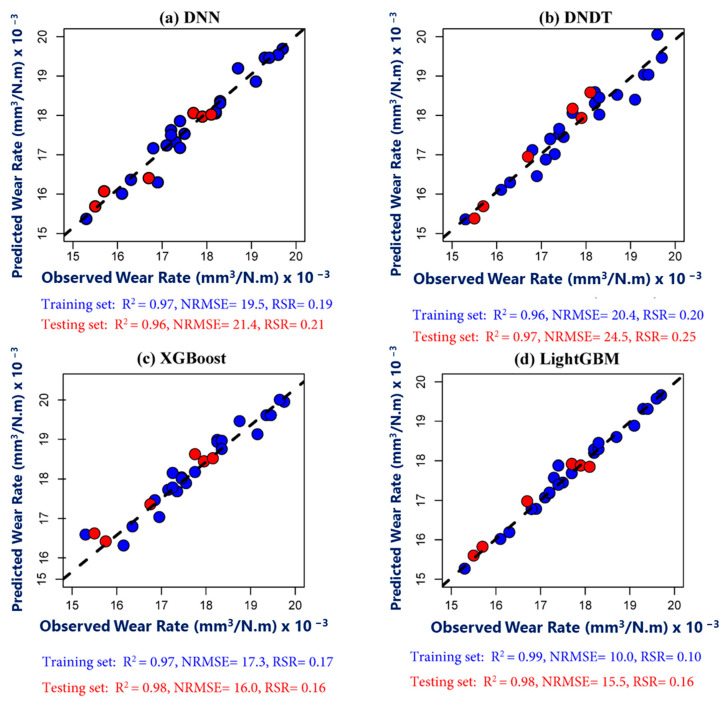
Comparison of predicted wear rate performance in inner zone with (**a**) DNN, (**b**) DNDT, (**c**) XGBoost, and (**d**) LightGBM models against the observed values during training and testing phases.

The scatter distribution with respect to the statistical performance of predicting wear rate in the inner zone during the training and testing periods illustrated in Figure 18. It is clear to observe that all ML models performed and captured the observed values well. During the training period, the LightGBM model exhibited the highest accuracy compared to the other models, characterized by R^2^ = 0.99, NRMSE = 10, and RSR = 0.10. Meanwhile, the XGBoost and LightGBM models performed better than the DNDT and DNN models during the testing period with respect to R^2^ (0.98, 0.98), NRMSE (16.0, 15.5), and RSR = (0.16, 0.16). In general, all models showed a slight difference between the performance during the two periods, suggested that the hyperparameter techniques reduced the overfitting of the prediction. Table 7 summarizes the performance metrics R^2^, NRMSE, and RSR of each model in volume loss prediction. It is reported that an R^2^ value between 0.7 and 0.9 is considered acceptable for a model. However, if the R^2^ value exceeds 0.9, then the model is considered excellent [67,68]. According to Table 7, the R^2^ metric of the validation dataset ranged from 0.92 to 0.98. The lowest R^2^ value (0.92) was achieved by DNDT in the middle zone, while the highest R^2^ value (0.98) was obtained by LightGBM in the inner zone.

### 4.2. ML Models’ Performance in Middle Zone

**Figure 19:**
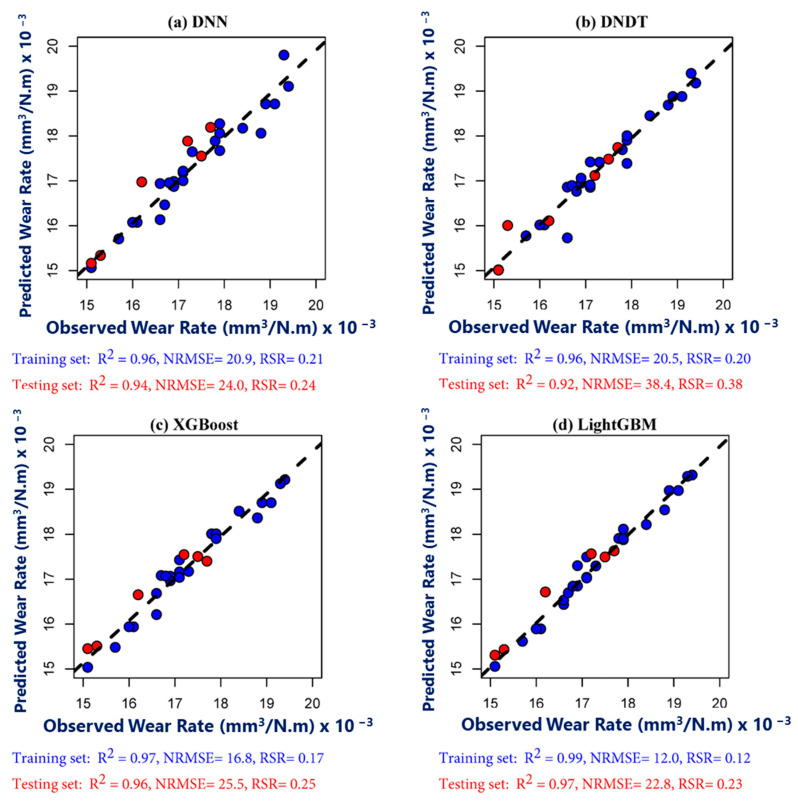
Comparison of predicted wear rate performance in middle zone with (**a**) DNN, (**b**) DNDT, (**c**) XGBoost, and (**d**) LightGBM models against the observed values during training and testing phases.

Figure 19 shows the scatter distribution of the predicted wear rate across the middle zone against the observed values during the training and testing phases. It is clear that the prediction performance during the two periods was excellent. Lower performance observed through DNN model during training period characterized by R^2^ = 0.96, NRMSE = 20.9, and RSR = 0.21. However, DNDT model had a lower performance during testing phase than other models, while showed comparable performance during testing period to DNN model. It obviously that the LightGBM model reflect exceptional accuracy than other models during testing phase, which indicates by R^2^ = 0.97, NRMSE = 22.8, and RSR = 0.23. Furthermore, the XGBoost model reflect higher potential than DNN and DNDT model for prediction wear rate in middle zone. Recent investigation reported that XGBoost model is capable to predict wear performance than Support Vector Regressor (SVR) and Multi-Layer Perceptron (MLP) [67].

### 4.3. ML Models’ Performance in Outer Zone

**Figure 20:**
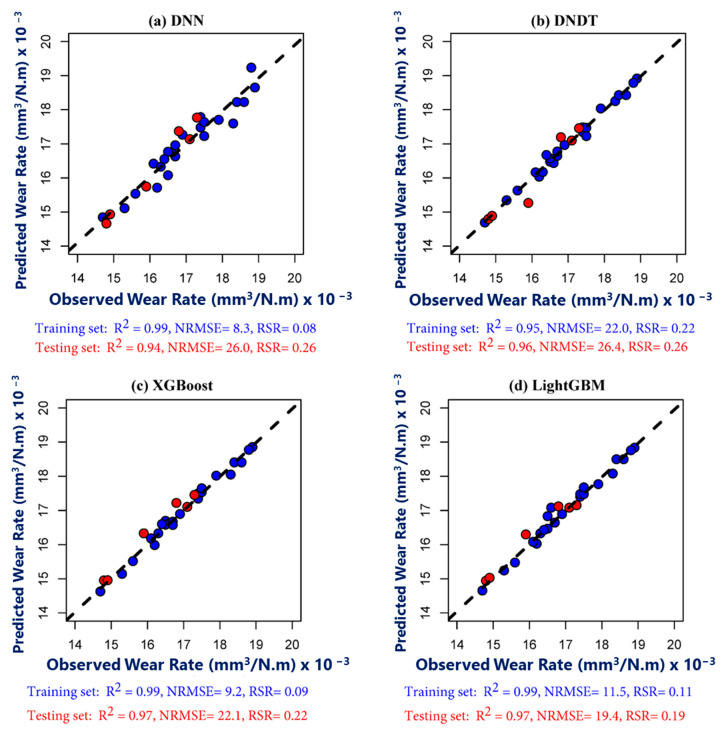
Comparison of predicted wear rate performance in outer zone with (**a**) DNN, (**b**) DNDT, (**c**) XGBoost, and (**d**) LightGBM models against the observed values during training and testing phases.

The predicting of wear rate a cross outer zone using four ML models illustrated in Figure 20. Overall, DNN and LightGBM models predict wear rate with highest accuracy than other models during training and testing periods, respectively. One conceivable rationale for this phenomenon may stem from the dataset’s modest scale and limited dimensionality. In such instances, a plausible contributing factor could be the challenge of overfitting, wherein inadequate data leads to difficulties in generalizing findings effectively [68,69]. Meanwhile, DNN model showed comparable performance during testing period to DNDT model. During testing phase, statistical performance of XGBoost model indicates by R^2^ = 0.97, NRMSE = 22.1, and RSR = 0.22, whereas LightGBM model indicates by R^2^ = 0.97, NRMSE = 19.4, and RSR = 0.19. Interestingly, tree-based ML models (XGBoost and LightGBM) produced more successful results than deep neural models (DNN and DNDT). This discovery aligns with the latest findings reported by Aydin et al. [70].

**Figure 21:**
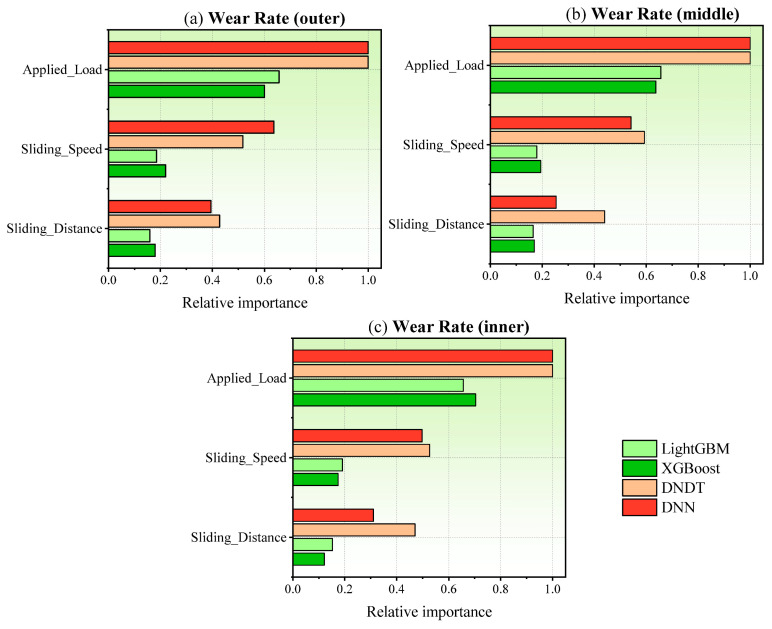
The feature importance of explanatory variables for the prediction of wear rate across three different zones.

Figure 21 presents the feature importance analysis of three explanatory input parameters namely, applied load, sliding speed, and sliding distance in the estimation of wear rates across three distinct zones, through employing four different ML models. It is evident from the graph that each variable contributes to the prediction of wear rates. Notably, the applied load emerges as the most influential variable in the prediction process, corroborating findings from prior literature, where load has been consistently identified as a primary determinant of wear rate prediction using machine learning methodologies [71,72,73]. Furthermore, sliding speed and distance also demonstrate significant importance in the prediction model. In other words, NDNT and DNN models reflects a high level of feature importance for all input variables.

The proposed machine learning method offers several notable advantages when compared to previous studies mentioned in the introduction. In terms of accuracy, the machine learning models, particularly LightGBM and XGBoost, significantly outperformed traditional empirical and analytical approaches used in past research. For example, the LightGBM model achieved superior accuracy, with an R^2^ of 0.99 during training and 0.98 during testing for the inner zone, a substantial improvement over the moderate accuracy levels reported by methods such as those used by Sharma et al. [74] and Choudhary et al. [75]. Another key advantage lies in the ability of these machine learning models to handle complex, multidimensional data. While earlier studies primarily focused on simpler factors influencing wear, such as applied load and sliding speed, the proposed method efficiently incorporated multiple explanatory variables, including load, speed, and sliding distance. This was achieved through the feature importance analysis, which demonstrated the significant role of applied load in predicting wear rates, providing a level of insight not afforded by traditional methods. Moreover, the proposed machine learning approach demonstrated enhanced generalization and reduced overfitting, overcoming a common limitation in earlier works, where models were often tailored to specific zones and struggled to generalize across others. The hyperparameter tuning techniques employed by the machine learning models contributed to the minimal performance differences between training and testing periods, a marked improvement over previous zone-specific model [76]. Interestingly, the tree-based models in this study (LightGBM and XGBoost) outperformed the deep learning models (DNN, DNDT), especially when dealing with a modest-sized dataset. While deep learning models in prior studies often struggled with overfitting and required extensive datasets, the tree-based models in this study proved more robust, delivering exceptional accuracy even in the outer zone, where LightGBM achieved an R^2^ of 0.97. This indicates a shift from the reliance on deep learning models seen in previous works, such as Sourabh et al. [27], toward more efficient tree-based approaches. Finally, the proposed machine learning models offered greater interpretability through the feature importance analysis, which identified key factors influencing wear rates. This contrasts with the more limited interpretative capacity of traditional empirical models, such as those used by Sharma et al. [74], which often did not provide detailed insights into the importance of individual input variables. In summary, the proposed machine learning approach marks a substantial leap forward from earlier research, providing heightened predictive precision, improved management of intricate data, enhanced generalization, and increased interpretive clarity.

Overall, the developed models offer a rapid and efficient means to assess the suitability of functionally graded magnesium composites reinforced with eggshell particles for specific applications based on predefined requirements, potentially reducing the need for extensive additional tests. By inputting desired application criteria into the model, such as wear resistance thresholds and load-bearing capacities, the predictive capabilities of the machine learning algorithms can swiftly evaluate whether the composite material meets these criteria. This evaluation process streamlines material assessment by leveraging the data-driven insights provided by the models, enabling decision-makers to expedite the selection of materials best suited for the intended applications. Thus, the developed models, correlating wear test parameters such as applied load, sliding speed, and sliding distance, with wear properties, aim to optimize boosting algorithms for rapid material assessment, aiding in selecting materials based on predefined requirements without extensive testing, thereby enhancing industrial efficiency and decision-making processes.

## 5. Conclusions

In this study, functionally graded magnesium composites reinforced with eggshell particles were effectively manufactured through stir casting and horizontal centrifugal casting methods. The study focused on exploring wear performance and mechanisms in these samples, alongside developing machine learning models for wear prediction. Four unique machine learning algorithms were employed for correlating wear test parameters such as applied load, sliding speed, and sliding distance to forecast wear behavior, their efficacy assessed using a diverse dataset from multiple test operations. These models offer a swift and efficient approach to assess the suitability of functionally graded magnesium composites reinforced with eggshell particles for specific applications, potentially reducing the requirement for extensive additional tests. The key findings of the study include:

1. The incorporation of AZ91 chips and eggshell particles in the manufacture of the graded composite exemplifies an eco-friendly approach that aims to minimize waste and support environmentally conscious production methods. By repurposing AZ91 chips and incorporating eggshell particles, the study addresses the issue of waste generation in the magnesium industry. This sustainable approach aligns with the principles of the circular economy and environmental responsibility.
2. In the outer zone, there was a 25.26% increase in hardness over the inner zone due to the particle gradient, with wear resistance improving by 19.8% compared to the inner zone.
3. The wear resistance of the graded composite displayed a decrease as the applied load and sliding distance increased. Notably, the impact of an increase in applied load on reducing the wear resistance was more significant compared to the effect of sliding distance. Furthermore, the wear rate initially showed an increase with sliding speed, reaching its peak at 2.5 m/s. However, at speeds exceeding 2.5 m/s, the wear rate demonstrated a slight reduction.
4. Among the machine learning models developed, the tree-based ML model outperformed the deep neural-based ML models in predicting the wear rate. The LightGBM model demonstrated the highest accuracy in predicting the testing set across the three zones.
5. The importance of input features was analyzed using the four ML models. It was observed that the prediction of wear rate across the three zones was primarily influenced by the applied load parameter.

These findings contribute to the understanding of the wear performance and wear mechanisms of functionally graded magnesium composites reinforced with eggshell particles. The application of machine learning models provides a predictive tool for assessing wear performance, with the tree-based ML model, specifically the LightGBM model, showing the best performance in this study. Moving forward, addressing current challenges such as enhancing the model’s robustness in diverse operational conditions and scaling up production efficiency could be pivotal. Despite the successful development and application of functionally graded magnesium composites reinforced with eggshell particles, there are several challenges to address in future research. One major challenge is the need for more comprehensive datasets to improve the robustness and generalizability of the machine learning models. The current study demonstrated the effectiveness of tree-based models like LightGBM, but the performance of deep neural network models was less impressive, suggesting that more advanced or fine-tuned deep learning techniques might be necessary. Another challenge lies in the scalability of the manufacturing process. While the use of AZ91 chips and eggshell particles is an eco-friendly approach, the uniformity and consistency of the composite materials across larger-scale production need to be ensured. Additionally, understanding the long-term wear behavior under varying environmental conditions is crucial for real-world applications, which requires extensive testing beyond the conditions studied here.

Future research should focus on addressing these challenges by expanding the dataset used for training machine learning models, incorporating a wider range of operating conditions, and possibly integrating more advanced algorithms that can better capture the complexities of wear mechanisms. The exploration of hybrid machine learning models that combine the strengths of both tree-based and deep learning approaches could also be beneficial. In terms of material development, improving the stir casting and centrifugal casting processes to enhance the uniformity and mechanical properties of the composites on a larger scale will be important. Further studies could investigate the wear performance of these composites under different environmental conditions, such as varying temperatures and corrosive environments, to better understand their applicability in diverse real-world scenarios. Ultimately, investigating additional eco-friendly reinforcement materials or their combinations could enhance the sustainability and efficacy of these composites. Future endeavors might encompass exploring innovative reinforcement materials like rice husk ash, groundnut shell ash, and coconut shell ash, refining manufacturing techniques, and expanding research into practical applications to propel the domain of functionally graded composites and predictive wear analysis forward.

## Figures and Tables

**Table 1:** Properties of AZ91 magnesium alloy used in this study.

Properties	Density at 20 °C (g/cm^3^)	Melting Point (°C)	Thermal Expansion Coefficient (µm/m°C)	Poisson Ratio	Hardness (HV)	Tensile Strength (MPa)	Elongation (%)	Young’s Modulus (GPa)
AZ91 Alloy	1.81	≥421	26	0.35	65	165	4.72	45

**Table 2:** Wear test parameters and their levels.

Factors	Levels							Units
Applied Load	5	10	15	20	25	30	35	N
Sliding Speed	0.5	1	1.5	2	2.5	3	3.5	m/s
Sliding Distance	500	1000	1500	2000	2500	3000	3500	m

**Table 3:** The experimental values along with the corresponding wear rate responses.

Run	Factors			Responses		
	Applied Load (N)	Sliding Speed (m/s)	Sliding Distance (m)	Wear Rate (Outer) (mm^3^/N.m)	Wear Rate (Middle) (mm^3^/N.m)	Wear Rate (Inner) (mm^3^/N.m)
1	20	2	2000	0.0171	0.0175	0.0179
2	20	2	2000	0.0171	0.0175	0.0179
3	5	1.5	3000	0.0159	0.0162	0.0167
4	25	3.5	2000	0.0168	0.0172	0.0177
5	5	1.5	500	0.0148	0.0151	0.0155
6	10	3.5	1000	0.0149	0.0153	0.0157
7	20	2	2000	0.0171	0.0175	0.0179
8	35	0.5	1500	0.0173	0.0177	0.0181
9	5	0.5	2000	0.0147	0.0151	0.0153
10	5	1.5	500	0.0148	0.0151	0.0155
11	10	2.5	1500	0.0162	0.0166	0.0169
12	20	2	2000	0.0171	0.0175	0.0179
13	10	1.5	1000	0.0153	0.0157	0.0161
14	35	1	3500	0.0189	0.0194	0.0197
15	20	2	2000	0.0171	0.0175	0.0179
16	10	3.5	1000	0.0149	0.0153	0.0157
17	20	2	2000	0.0171	0.0175	0.0179
18	5	3	3500	0.0161	0.0166	0.0168
19	15	0.5	3500	0.0165	0.0169	0.0173
20	5	0.5	2000	0.0147	0.0151	0.0153
21	20	2	2000	0.0171	0.0175	0.0179
22	20	2	2000	0.0171	0.0175	0.0179
23	15	2	2000	0.0166	0.0171	0.0174
24	20	0.5	500	0.0156	0.0161	0.0163
25	20	2	2000	0.0171	0.0175	0.0179
26	35	3	500	0.0174	0.0179	0.0182
27	20	2	2000	0.0171	0.0175	0.0179
28	20	0.5	500	0.0156	0.016	0.0163
29	20	2	2000	0.0171	0.0175	0.0179
30	20	2	2000	0.0171	0.0175	0.0179
31	20	2	2000	0.0171	0.0175	0.0179
32	20	2.5	3500	0.0183	0.0188	0.0191
33	20	2	2000	0.0171	0.0175	0.0179
34	20	2	2000	0.0171	0.0175	0.0179
35	35	2	2000	0.0184	0.0189	0.0193
36	20	2	2000	0.0171	0.0175	0.0179
37	20	2	2000	0.0171	0.0175	0.0179
38	35	1	500	0.0174	0.0178	0.0182
39	35	3.5	3500	0.0188	0.0193	0.0196
40	20	2	1000	0.0167	0.0171	0.0175
41	35	1	1000	0.0175	0.0179	0.0183
42	20	1	1500	0.0163	0.0167	0.0171
43	20	1.5	1500	0.0165	0.0169	0.0172
44	25	1.5	1000	0.0169	0.0173	0.0177
45	25	2.5	1500	0.0175	0.0179	0.0183
46	20	3.5	2000	0.0164	0.0168	0.0172
47	20	1.5	2000	0.0167	0.0171	0.0174
48	30	2	2000	0.0179	0.0184	0.0187
49	35	2	2000	0.0186	0.0191	0.0194

**Table 4:** The parameter range used in hyperparameter tuning of XGBoost and LightGBM models and their optimal value for predicting wear rate in different zones (inner, middle, & outer).

Model	Parmeter	Range	Optimized Value		
			Inner	Middel	Outer
**XGB**	eta	[0.001, 0.2]	0.003	0.023	0.05
	max_depth	[3.0, 9.0]	4.0	4.0	4.0
	n_estimators	[300, 500]	467.0	394.0	351.0
	learning_rate	[0.001, 0.3]	0.137	0.184	0.21
	gamma	[0.001, 0.3]	0.14	0.058	0
	subsample	[0.5, 1.0]	0.573	0.979	0.7
	colsample_bytree	[0.5, 1.0]	0.925	0.643	0.86
	min_child_weight	[3.0, 9.0]	4.0	4.0	4.0
**LightGBM**	max_depth	[3.0, 9.0]	4.0	4.0	4.0
	num_leaves	[10.0, 500]	485.0	485.0	485.0
	min_data_in_leaf	[1.0, 50.0]	4.0	4.0	4.0
	feature_fraction	[0.01, 1.0]	0.06	0.06	0.06
	bagging_fraction	[0.01, 1.0]	0.64	0.64	0.64
	learning_rate	[0.01, 0.03]	0.26	0.26	0.26

**Table 5:** The parameter used in training DNDT and DNN models for predicting wear rate in different zones (inner, middle, and outer).

Model	Parameter	Value
DNDT	hiddenLayerUnits	c (150, 50)
	activation	c (“sigmoid”, “relu”)
	reluLeak	0.001
	modelType	regression
	iterations	5000
	eta	0.001
	optimiser	Adam
DNN	hiddenLayerUnits	c(150, 100)
	activation	c (“sigmoid”, “relu”)
	reluLeak	0.001
	modelType	regression
	iterations	5000
	eta	0.1
	optimiser	Adam

**Table 6:** Summary of wear mechanisms observed in the graded composite in different zones.

Wear Parameters		Pin Zones	Wear Mechanisms			
Load (N)	Speed (m/s)		Abrasion	Oxidation	Delamination	Melting
**5**	0.5	outer	√ ^*^	√		
		middle	√	√		
		inner	√√	√	√	
	2.5	outer	√	√		
		middle	√	√√		
		inner	√√	√√	√	
	3.5	outer	√	√		
		middle	√	√	√	
		inner	√√	√√		
**35**	0.5	outer		√	√	
		middle		√√	√	
		inner			√	√
	2.5	outer				√
		middle				√√
		inner				√√√
	3.5	outer		√	√	
		middle			√	√
		inner			√	√√

* The relative extent of each wear mechanism: √: slight; √√: moderate; √√√: heavy.

**Table 7:** The performance comparison of machine learning models on the validation data, considering various performance metrics.

Target	Models	Training			Testing		
		R^2^	NRMSE	RSR	R^2^	NRMSE	RSR
Wear Rate (Outer)	XGBoost	0.99	9.2	0.09	0.97	22.1	0.22
	DNDT	0.95	22.0	0.22	0.96	26.4	0.26
	DNN	0.99	8.3	0.08	0.94	26.0	0.26
	LightGBM	0.99	11.5	0.11	0.97	19.4	0.19
Wear Rate (Middle)	XGBoost	0.97	16.8	0.17	0.96	25.5	0.25
	DNDT	0.96	20.5	0.20	0.92	38.4	0.38
	DNN	0.96	20.9	0.21	0.94	24.0	0.24
	LightGBM	0.99	12.0	0.12	0.97	22.8	0.23
Wear Rate (Inner)	XGBoost	0.97	17.3	0.17	0.98	16.0	0.16
	DNDT	0.96	20.4	0.20	0.97	24.5	0.25
	DNN	0.97	19.5	0.19	0.96	21.4	0.21
	LightGBM	0.99	10.0	0.10	0.98	15.5	0.16

## Data Availability

The original contributions presented in the study are included in the article, further inquiries can be directed to the corresponding authors.

## References

[1] Saleh B., Fathi R., Zhang L., Yu Z., Liu S., Zhao L. (2024). Enhancing mechanical and wear performances of magnesium matrix composites using low-cost squid quill ash. Compos. Part B Eng..

[2] Asgari A., Sedighi M., Krajnik P. (2019). Magnesium alloy-silicon carbide composite fabrication using chips waste. J. Clean. Prod..

[3] Baffari D., Buffa G., Campanella D., Fratini L., Reynolds A.P. (2017). Process mechanics in Friction Stir Extrusion of magnesium alloys chips through experiments and numerical simulation. J. Manuf. Process..

[4] Paraskevas D., Dadbakhsh S., Vleugels J., Vanmeensel K., Dewulf W., Duflou J.R. (2016). Solid state recycling of pure Mg and AZ31 Mg machining chips via spark plasma sintering. Mater. Des..

[5] Seetharaman S., Subramanian J., Singh R.A., Wong W.L.E., Nai M.L.S., Gupta M. (2022). Mechanical Properties of Sustainable Metal Matrix Composites: A Review on the Role of Green Reinforcements and Processing Methods. Technologies.

[6] Parande G., Manakari V., Kopparthy S.D.S., Gupta M. (2018). Utilizing Low-Cost Eggshell Particles to Enhance the Mechanical Response of Mg–2.5Zn Magnesium Alloy Matrix. Adv. Eng. Mater..

[7] Ramanujam N., Muthukumaran S., Nagesawara Rao B., Ramarao M., Mangrulkar A.L., Ashraff Ali K.S., Pugazhendhi L., Markos M. (2022). Experimental Investigations on Mechanical Properties of AZ31/Eggshell Particle-Based Magnesium Composites. Adv. Mater. Sci. Eng..

[8] Srivastava A.K., Dwivedi S., Nag A., Kumar D., Dixit A.R., Hloch S. (2023). Microstructural, mechanical and tribological performance of a magnesium alloy AZ31B/Si3N4/eggshell surface composite produced by solid-state multi-pass friction stir processing. Mater. Chem. Phys..

[9] Demirdal S., Aydın F. (2022). The influence of low-cost eggshell on the wear and electrochemical corrosion behaviour of novel pure Mg matrix composites. Mater. Chem. Phys..

[10] Fathi R., Wei H., Saleh B., Radhika N., Jiang J., Ma A., Ahmed M.H., Li Q., Ostrikov K.K. (2022). Past and present of functionally graded coatings: Advancements and future challenges. Appl. Mater. Today.

[11] Sam M., Radhika N., Saleh B. (2023). Improving reciprocal tribo-mechanical performance and corrosion resistance using carbide ceramics: T6 thermal-treated A333 mono-hybrid functionally graded composites. Mater. Today Chem..

[12] Nguyen V.-H., Trinh M.-C., Jun H. (2024). Fracture behavior of thermal mismatch in functionally graded materials using phase-field modeling. Eng. Fract. Mech..

[13] Deng J., Ren X., Lin H., Hu L., Bai Y., Yu X., Mo J., Zhang Q., Kang F., Li B. (2024). Functionally gradient materials for sustainable and high-energy rechargeable lithium batteries: Design principles, progress, and perspectives. J. Energy Chem..

[14] Abdelaal O., Hengsbach F., Schaper M., Hoyer K. (2022). LPBF Manufactured Functionally Graded Lattice Structures Obtained by Graded Density and Hybrid Poisson’s Ratio. Materials.

[15] Saleh B., Ji B., Fathi R., Guo S., Ahmed M.H. (2022). Utilization of machining chips waste for production of functionally gradient magnesium matrix composites. J. Mater. Process. Tech..

[16] Saleh B., Ma A., Fathi R., Radhika N., Ji B., Jiang J. (2022). Wear Characteristics of Functionally Graded Composites Synthesized from Magnesium Chips Waste. Tribol. Int..

[17] Saleh B., Fathi R., Radhika N., Yu Z., Liu S., Zhang L. (2024). Effect of yttrium oxide on microstructure and mechanical properties of functionally graded magnesium matrix composites. J. Alloys Compd..

[18] Saleh B., Ma A., Fathi R., Radhika N., Yang G. (2023). Optimized mechanical properties of magnesium matrix composites using RSM and ANN. Mater. Sci. Eng. B.

[19] Saleh B., Fathi R., Abdalla M.A.A., Radhika N., Ma A., Jiang J. (2023). Optimization and Characterization of Centrifugal-Cast Functionally Graded Al-SiC Composite Using Response Surface Methodology and Grey Relational Analysis. Coatings.

[20] Arifuzzaman M., Hasan M.R., Toma T.J., Hassan S.B., Paul A.K. (2023). An Advanced Decision Tree-Based Deep Neural Network in Nonlinear Data Classification. Technologies.

[21] Schmidhuber J. (2015). Deep learning in neural networks: An overview. Neural Netw..

[22] Elzain H.E., Abdalla O.A., Abdallah M., Al-Maktoumi A., Eltayeb M., Abba S.I. (2024). Innovative approach for predicting daily reference evapotranspiration using improved shallow and deep learning models in a coastal region: A comparative study. J. Environ. Manag..

[23] Abdallah M., Mohammadi B., Nasiri H., Katipoğlu O.M., Abdalla M.A.A., Ebadzadeh M.M. (2023). Daily global solar radiation time series prediction using variational mode decomposition combined with multi-functional recurrent fuzzy neural network and quantile regression forests algorithm. Energy Rep..

[24] Wang N., Zhang G., Pang W., Ren L., Wang Y. (2021). Novel monitoring method for material removal rate considering quantitative wear of abrasive belts based on LightGBM learning algorithm. Int. J. Adv. Manuf. Technol..

[25] Mahmood J., Mustafa G., Ali M. (2022). Accurate estimation of tool wear levels during milling, drilling and turning operations by designing novel hyperparameter tuned models based on LightGBM and stacking. Measurement.

[26] Gao K., Chen H., Zhang X., Ren X.K., Chen J., Chen X. (2019). A novel material removal prediction method based on acoustic sensing and ensemble XGBoost learning algorithm for robotic belt grinding of Inconel 718. Int. J. Adv. Manuf. Technol..

[27] Sourabh K., Singh K., Kumar S., Singh K.K. (2022). Computational data-driven based optimization of tribological performance of graphene filled glass fiber reinforced polymer composite using machine learning approach. Mater. Today Proc..

[28] Xu Q., Ma A., Saleh B., Li Y., Yuan Y., Jiang J., Ni C. (2020). Enhancement of strength and ductility of SiCp/AZ91 composites by RD-ECAP processing. Mater. Sci. Eng. A.

[29] Turan M.E., Aydin F. (2022). Wear and corrosion properties of low-cost eggshell-reinforced green AZ91 matrix composites. Can. Metall. Q..

[30] (2005). Method for Wear Testing with a Pin-on-Disk Apparatus.

[31] Touzani S., Granderson J., Fernandes S. (2018). Gradient boosting machine for modeling the energy consumption of commercial buildings. Energy Build..

[32] Friedman J.H. (2001). Greedy function approximation: A gradient boosting machine. Ann. Stat..

[33] Ghafarian F., Wieland R., Lüttschwager D., Nendel C. (2022). Application of extreme gradient boosting and Shapley Additive explanations to predict temperature regimes inside forests from standard open-field meteorological data. Environ. Model. Softw..

[34] Ahmed N.K., Atiya A.F., El Gayar N., El-Shishiny H. (2010). An empirical comparison of machine learning models for time series forecasting. Econom. Rev..

[35] Chen T., Guestrin C. XGBoost: A scalable tree boosting system. Proceedings of the KDD ’16: 22nd ACM SIGKDD International Conference on Knowledge Discovery and Data Mining.

[36] Yang Y., Morillo I.G., Hospedales T.M. Deep Neural Decision Trees. Proceedings of the ICML Workshop on Human Interpretability in Machine Learning (WHI 2018).

[37] Quinlan J.R. (1994). Book Review: C4. 5: Programs for Machine Learning.

[38] Norouzi M., Collins M., Johnson M.A., Fleet D.J., Kohli P. Efficient Non-greedy Optimization of Decision Trees. Proceedings of the Advances in Neural Information Processing Systems.

[39] Dougherty J., Kohavi R., Sahami M. Supervised and unsupervised discretization of continuous features. Proceedings of the Twelfth International Conference on Machine Learning.

[40] Hanna A.M., Ural D., Saygili G. (2007). Neural network model for liquefaction potential in soil deposits using Turkey and Taiwan earthquake data. Soil Dyn. Earthq. Eng..

[41] Dahl G.E., Sainath T.N., Hinton G.E. Improving deep neural networks for LVCSR using rectified linear units and dropout. Proceedings of the 2013 IEEE International Conference on Acoustics, Speech and Signal Processing.

[42] Tripathy R.K., Bilionis I. (2018). Deep UQ: Learning deep neural network surrogate models for high dimensional uncertainty quantification. J. Comput. Phys..

[43] Wang J., Wang H., Wang X., Chang H. (2020). Predicting Drug-target Interactions via FM-DNN Learning. Curr. Bioinform..

[44] Snoek J., Larochelle H., Adams R.P. Practical Bayesian Optimization of Machine Learning Algorithms. Proceedings of the Advances in Neural Information Processing Systems.

[45] Wilson S. (2021). ParBayesianOptimization: Parallel Bayesian Optimization of Hyperparameters, R package version 1.2.4. https://cran.r-project.org/package=ParBayesianOptimization.

[46] Abdallah M., Mohammadi B., Zaroug M.A., Omer A., Cheraghalizadeh M., Eldow M.E., Duan Z. (2022). Reference evapotranspiration estimation in hyper-arid regions via D-vine copula based-quantile regression and comparison with empirical approaches and machine learning models. J. Hydrol. Reg. Stud..

[47] Stephen K.D., Kazemi A. (2014). Improved normalization of time-lapse seismic data using normalized root mean square repeatability data to improve automatic production and seismic history matching in the Nelson field. Geophys. Prospect..

[48] Huang S., Abbas A. (2020). Effects of tungsten disulfide on microstructure and mechanical properties of AZ91 magnesium alloy manufactured by stir casting. J. Alloys Compd..

[49] Fathi R., Ma A., Saleh B., Xu Q., Jiang J. (2020). Investigation on mechanical properties and wear performance of functionally graded AZ91-SiCp composites via centrifugal casting. Mater. Today Commun..

[50] Saleh B., Ahmed M. (2020). Development of Functionally Graded Tubes Based on Pure Al/Al_2_O_3_ Metal Matrix Composites Manufactured by Centrifugal Casting for Automotive Applications. Met. Mater. Int..

[51] Saleh B., Jiang J., Fathi R., Xu Q., Li Y., Ma A. (2021). Influence of gradient structure on wear characteristics of centrifugally cast functionally graded magnesium matrix composites for automotive applications. Arch. Civ. Mech. Eng..

[52] Xiao H., Ma G., Ye J., He Y. (2021). Preparation of graphene reinforced AZ31B magnesium-based composites by stirring casting. Vacuum.

[53] Mohammadi H., Emamy M., Hamnabard Z. (2019). The Statistical Analysis of Tensile and Compression Properties of the As-Cast AZ91-X % B4C Composites. Int. J. Met..

[54] Sam M., Radhika N., Saleh B. (2022). Influence of boride, oxide, and carbide ceramics as secondary reinforcement in T6-A333 functionally graded hybrid composites. Ceram. Int..

[55] Saleh B., Jiang J., Fathi R., Xu Q., Wang L., Ma A. (2020). Study of the microstructure and mechanical characteristics of AZ91–SiCp composites fabricated by stir casting. Arch. Civ. Mech. Eng..

[56] Sahoo B., Panigrahi S. (2019). Development of wear maps of in-situ TiC+TiB2 reinforced AZ91 Mg matrix composite with varying microstructural conditions. Tribol. Int..

[57] Xu Q., Ma A., Saleh B., Fathi R., Li Y., Jiang J., Ni C. (2020). Dry Sliding Wear Behavior of AZ91 Alloy Processed by Rotary-Die Equal Channel Angular Pressing. J. Mater. Eng. Perform..

[58] Selvam B., Marimuthu P., Narayanasamy R., Anandakrishnan V., Tun K.S., Gupta M., Kamaraj M. (2014). Dry sliding wear behaviour of zinc oxide reinforced magnesium matrix nano-composites. Mater. Des..

[59] Kumar A., Kumar S., Mukhopadhyay N.K., Yadav A., Kumar V. (2021). Effect of Variation of SiC Reinforcement on Wear Behaviour of AZ91 Alloy. Materials.

[60] Shen M., Zhu X., Han B., Ying T., Jia J. (2021). Dry sliding wear behaviour of AZ31 Magnesium alloy strengthened by nanoscale SiCp. J. Mater. Res. Technol..

[61] Lim C., Lim S., Gupta M. (2003). Wear behaviour of SiCp-reinforced magnesium matrix composites. Wear.

[62] Wei T.Z., Shamsuri S.R.B., Chang S.Y., Rashid M.W.A., Ahsan Q. (2013). Effect of sliding velocity on wear behavior of magnesium composite reinforced with SiC and MWCNT. Procedia Eng..

[63] Xiao P., Gao Y., Xu F., Yang C., Li Y., Liu Z., Zheng Q. (2018). Tribological behavior of in-situ nanosized TiB2 particles reinforced AZ91 matrix composite. Tribol. Int..

[64] Saleh B., Jiang J., Xu Q., Fathi R., Ma A., Li Y., Wang L. (2021). Statistical Analysis of Dry Sliding Wear Process Parameters for AZ91 Alloy Processed by RD-ECAP Using Response Surface Methodology. Met. Mater. Int..

[65] Taylor P., Yigezu B.S., Jha P.K., Mahapatra M.M. (2013). Effect of Sliding Distance, Applied Load, and Weight Percentage of Reinforcement on the Abrasive Wear Properties of In Situ Synthesized Al-12 %Si/TiC Composites. Tribol. Trans..

[66] García-Rodríguez S., Torres B., Maroto A., López A., Otero E., Rams J. (2017). Dry sliding wear behavior of globular AZ91 magnesium alloy and AZ91/SiCp composites. Wear.

[67] Hasan M.S., Wong T., Rohatgi P.K., Nosonovsky M. (2022). Analysis of the friction and wear of graphene reinforced aluminum metal matrix composites using machine learning models. Tribol. Int..

[68] Matrenin P., Safaraliev M., Dmitriev S., Kokin S., Eshchanov B., Rusina A. (2022). Adaptive ensemble models for medium-term forecasting of water inflow when planning electricity generation under climate change. Energy Rep..

[69] Marian M., Tremmel S. (2021). Current trends and applications of machine learning in tribology—A review. Lubricants.

[70] Aydin F., Durgut R., Mustu M., Demir B. (2023). Prediction of wear performance of ZK60/CeO_2_ composites using machine learning models. Tribol. Int..

[71] Borjali A., Monson K., Raeymaekers B. (2019). Predicting the polyethylene wear rate in pin-on-disc experiments in the context of prosthetic hip implants: Deriving a data-driven model using machine learning methods. Tribol. Int..

[72] Kolev M., Drenchev L., Petkov V., Dimitrova R., Kovacheva D. (2023). Open-Cell AlSn6Cu-SiC Composites: Fabrication, Dry-Sliding Wear Behavior, and Machine Learning Methods for Wear Prediction. Materials.

[73] Kolev M. (2023). XGB-COF: A machine learning software in Python for predicting the friction coefficient of porous Al-based composites with Extreme Gradient Boosting [Formula presented]. Softw. Impacts.

[74] Sharma V., Sharma S., Verma O.P., Bhardwaj B., Sharma T.K., Pachauri N. (2021). Prediction and optimization of abrasive wear loss of ultrahigh strength martensitic steel using response surface methodology, Harris Hawk and artificial neural network. Int. J. Syst. Assur. Eng. Manag..

[75] Choudhary L., Chhotani P., Menghani J. (2019). Study on Wear Behaviour on Hardfacing Alloy. Trans. Indian Inst. Met..

[76] Huang S., Adityawardhana Y. (2023). Predicting Mechanical Properties of Magnesium Matrix Composites with Regression Models by Machine Learning. J. Compos. Sci..

